# CB1R activation in nucleus accumbens core promotes stress-induced reinstatement of cocaine seeking by elevating extracellular glutamate in a drug-paired context

**DOI:** 10.1038/s41598-021-92389-4

**Published:** 2021-06-21

**Authors:** Andrea S. Guzman, Maria P. Avalos, Laura N. De Giovanni, Pia V. Euliarte, Marianela A. Sanchez, Bethania Mongi-Bragato, Daiana Rigoni, Flavia A. Bollati, Miriam B. Virgolini, Liliana M. Cancela

**Affiliations:** 1 Departamento de Farmacología, Facultad de Ciencias Químicas, Universidad Nacional de Córdoba, X5000HUA Córdoba, Argentina; 2 Instituto de Farmacología Experimental de Córdoba (IFEC-CONICET), X5000HUA Córdoba, Argentina

**Keywords:** Neurochemistry, Addiction, Motivation, Addiction, Stress and resilience, Classical conditioning, Extinction, Reward, Stress and resilience, Neurotransmitters

## Abstract

Preclinical models of stress-induced relapse to drug use have shown that the dysregulation of glutamatergic transmission within the nucleus accumbens (NA) contributes notably to the reinstatement of cocaine-seeking behavior in rodents. In this sense, there has been increasing interest in the cannabinoid type-1 receptor (CB1R), due to its crucial role in modulating glutamatergic neurotransmission within brain areas involved in drug-related behaviors. This study explored the involvement of CB1R within the NA subregions in the restraint stress-induced reinstatement of cocaine-conditioned place preference (CPP), as well as in the regulation of glutamatergic transmission, by using a pharmacological approach and the in vivo microdialysis sampling technique in freely moving rats. CB1R blockade by the antagonist/inverse agonist AM251 (5 nmol/0.5 μl/side) or CB1R activation by the agonist ACEA (0.01 fmol/0.5 μl/side), prevented or potentiated restraint stress-induced reinstatement of cocaine-CPP, respectively, after local administration into NAcore, but not NAshell. In addition, microdialysis experiments demonstrated that restraint stress elicited a significant increase in extracellular glutamate in NAcore under reinstatement conditions, with the local administration of AM251 or ACEA inhibiting or potentiating this, respectively. Interestingly, this rise specifically corresponded to the cocaine-associated CPP compartment. We also showed that this context-dependent change in glutamate paralleled the expression of cocaine-CPP, and disappeared after the extinction of this response. Taken together, these findings demonstrated the key role played by CB1R in mediating reinstatement of cocaine-CPP after restraint stress, through modulation of the context-specific glutamate release within NAcore. Additionally, CB1R regulation of basal extracellular glutamate was demonstrated and proposed as the underlying mechanism.

## Introduction

Stressful life events are an important factor that increases the risk of relapse to drug use in humans, since they can be harmful, uncontrollable and often unavoidable in everyday life^1,2^. This scenario has been modeled in rodents using different associative learning paradigms, such as self-administration and conditioned place preference (CPP), to induce ‘reinstatement’ of drug-seeking behavior through exposure to different stressors^3,4^. Consistent with a large body of research on therapeutic strategies for cocaine addiction^5^, different pharmacological treatments that target the glutamatergic system were able to prevent stress-induced reinstatement^6–9^. Specifically, a glutamatergic projection from the medial prefrontal cortex (mPFC) to the core subregion of the nucleus accumbens (NA) was reported to be critical for stress-induced reinstatement of cocaine seeking^10^, as is the case for cue- and drug-triggered reinstatement^11,12^. Stress causes changes in glutamate neurotransmission^13^, and it has been hypothesized that the recruitment of stress-responsive mechanisms contribute greatly to the persistence of drug addiction^14^.

The cannabinoid type-1 receptor (CB1R) has been increasingly studied in addiction research, due to its important role in modulating neurotransmission within brain areas primarily involved in drug-seeking behavior^15,16^. Although there is substantial evidence to suggest that CB1R mediates drug-, stress- and cue-induced reinstatement of cocaine-seeking behavior^17–24^, several works have found contrasting results^25–27^. For example, forced swim or restraint stress-induced reinstatement of extinguished cocaine-CPP was suppressed by the systemic administration of a CB1R antagonist/inverse agonist, AM251^24,25^. Nevertheless, no effect was observed on footshock stress-induced reinstatement of cocaine self-administration when systemic or intracerebroventricular administration of AM251 or SR141716A, another CB1R antagonist/inverse agonist, was used^17,26,27^. Differences in strain/species, behavioral approach (CPP vs. self-administration), different types of stressors (restraint vs. footshock), and doses and routes of administration of CB1R antagonists may contribute to the divergence^28^.

The only study to date that has explored pharmacologically the role of CB1R specifically in the NA in cocaine relapse reported inhibition of drug-induced reinstatement of cocaine self-administration in rats following local administration of AM251^18^. However, the authors did not distinguish between the core and shell subregions of the NA, which contribute in different ways to cocaine addiction (reviewed in^29^). Related to this, the present study aimed to systematically examine the differential role of the CB1R within the NA subregions in restraint stress-induced reinstatement of cocaine-CPP, taking into account previous findings from our laboratory that demonstrated the involvement of the glutamate N-methyl-D-aspartate receptors (NMDARs) within the NAcore, but not NAshell^6^.

The role of presynaptic CB1R in modulating different forms of glutamate plasticity has been reported in ex vivo electrophysiological studies^30^. In the canonical mechanism of action, the CB1R-mediated inhibition of glutamate release in the NA involves the activation of G protein-coupled inwardly-rectifying K^+^ channels^31^. Nevertheless, recent advances revealed a more complicated picture, and the inconsistencies with in vivo findings raise interesting questions. For instance, systemic administration of AM251 by itself has been shown to slowly raise extracellular glutamate levels in the NA, but prevented the rise induced by cocaine priming under reinstatement conditions^18^. Since reinstatement of cocaine seeking relies on increased presynaptic release of glutamate in the NAcore, that is facilitated by a preceding decrease in basal extracellular glutamate concentration after withdrawal from repeated cocaine^32,33^, it was hypothesized that the blockade of CB1R may reverse the latter effect to prevent synaptic potentiation and thereby reinstatement^18^.

Since the link between the endocannabinoid system, stress and reward has been well-established^34,35^, further research may help to understand the role of CB1R in stress- and drug-related behaviors. The present study, using intra-NA local microinjection of either a CB1R antagonist (AM251) or agonist (ACEA), tests the hypothesis that accumbal CB1R participates in the restraint stress-induced reinstatement of cocaine-seeking behavior through in vivo modulation of glutamate transmission in the NA. For this, we took advantage of combining the CPP paradigm with the in vivo microdialysis technique to monitor context-specific changes in accumbal extracellular glutamate in the absence of cocaine effects, and achieve a better understanding of the role of CB1R in modulating the expression of conditioned drug seeking. Moreover, we also studied the contribution of CB1R in regulating basal extracellular glutamate in vivo using the reverse microdialysis technique to locally and continuously perfuse ACEA into NAcore.

## Materials and methods

### Animals

Adult male Wistar rats bred in the animal facilities of the Institute of Experimental Pharmacology from Córdoba (IFEC-CONICET-UNC, Argentina) were used. These animals were maintained in groups in a temperature- and humidity-controlled room under a 12 h/12 h light/dark cycle, with free access to food and water. All animals were habituated to handling for two days prior to the beginning of the experiments. All experimental procedures were approved by the Institutional Animal Care and Use Committee of the Faculty of Chemical Sciences, National University of Córdoba (CICUAL permit number: Res. 680/2015), in accordance with the guidelines of the National Institutes of Health (*NIH*) *Guide for the Care and Use of Laboratory Animals*. This work also complied with the Animal Research: Reporting of In Vivo Experiments (ARRIVE) guidelines^36^.

### Drugs

Cocaine hydrochloride (Verardo Laboratory, Buenos Aires, Argentina) was dissolved in sterile saline (0.9%) at a concentration of 10 mg/mL for i.p. administration of a 10 mg/kg dose during the conditioning phase (see next section). ACEA (CB1R agonist) and AM251 (CB1R antagonist) were purchased from Sigma-Aldrich SA (St. Louis, MO, USA). ACEA was directly dissolved in sterile saline containing 0.1% dimethylsulfoxide (DMSO) (Sigma, USA). AM251 was first dissolved in pure DMSO and then saline in a 1:9 ratio. The 0.1% and 10% DMSO in saline solutions were used as the vehicle. The selection of doses used for intra-NA administration was based on previous studies (0.001 or 0.01 fmol/0.5 μl/side for ACEA^37^ and 2.5 or 5 nmol/0.5 μl/side for AM251^18^).

### Conditioning place-preference (CPP) apparatus and general procedure

The CPP paradigm was used as described in a previous study carried out at our laboratory^6^. The description of the CPP apparatus and the general procedure are given in Supplementary Materials and Methods. Some minor modifications to this procedure for each experiment are detailed below and further in the Results section. Briefly, the apparatus consisted of two boxes separated by a small corridor. Time spent in each chamber and locomotor activity were recorded using infrared photo beams and a computer interface (CPP v1.2, LIADE, Córdoba, Argentina). The CPP procedure consisted of the following phases: basal test, conditioning phase, conditioning test, extinction phase, extinction test and stress-induced reinstatement test. In the basal test (BT), each animal had free access to the entire apparatus for 15 min, and rats that showed unconditioned preference for either context were excluded (more than 66% of total time spent in main chambers, basal exclusion criterion). It is important to note that all the following tests were performed in the same way, but different criteria were considered. During the conditioning phase, animals were confined for 30 min to one chamber immediately after receiving cocaine (10 mg/kg i.p.) or to the opposite compartment after receiving saline. After eight days alternating between drug/saline sessions (4/4 days), the conditioning test (CT) was performed to confirm the expression of the conditioned preference for the cocaine-paired context. We included animals that spent more than 66% of total time in the cocaine-paired context. The extinction phase consisted of eight alternate saline associations with both contexts (confinement for 30 min, identical to conditioning) followed by the respective test (extinction test, ET). Animals that reached the extinction criterion (less than 66% of total time spent in the cocaine-paired context during the ET) were submitted the following day to the restraint stress-induced reinstatement test (RT). The protocol followed to evaluate the reinstatement of extinguished cocaine-CPP is described below according to each experiment.

### Experiment 1 and 2: effects of AM251 and ACEA in the NAcore or NAshell on restraint stress-induced reinstatement of cocaine-CPP

#### Surgery

The stereotaxic surgery to implant guide cannulae into the NA was performed after the fourth day of the extinction phase and animals were allowed 2–3 days to recover from the surgery, and then the extinction phase continued for the 4 remaining days. The procedure is described in Supplementary Materials and Methods. In brief, guide cannulae were implanted bilaterally, 2 mm dorsal to NAcore or NAshell in order to further use a microinjector projecting below its ventral end. Cannula placements were confirmed after finishing the experiments (the procedure is described in Supplementary Information and approximate locations are presented in each figure).

#### Experimental design

After animals reached the extinction criterion, they were divided into groups to observe the effects of AM251 and ACEA microinjection on stress-induced reinstatement of cocaine-CPP. For experiment 1, animals were divided into two groups to observe the effect of AM251 microinjected into the NAcore and NAshell. At the same time, subjects were assigned to six subgroups to determine the effects of two different doses of AM251 (2.5 or 5 nmol/0.5 μl/side) or vehicle (0.5 μl/side of 10% DMSO), received 5 min before 30 min of restraint stress (or no stress). For experiment 2, animals were divided into two groups to observe the effect of ACEA microinjected into the NAcore and NAshell. Subgroups received 0.001 or 0.01 fmol/0.5 μl/side of ACEA or 0.5 μl/side of 0.1% DMSO prior to 15 min of restraint stress (or no stress). The pharmacological specificity of ACEA for CB1R was confirmed by microinjecting 5 nmol/0.5 μl/side of AM251 (or vehicle) and 5 min after 0.01 fmol/0.5 μl/side of ACEA prior 15 min of restraint stress. Experimental groups are summarized in Supplementary table S1.

#### Microinfusion procedure and restraint stress-induced reinstatement

On the reinstatement test day, i.e. the day after ET, animals were first placed in the experimental room for 30 min in their home cages for habituation. After that, local microinjections were performed bilaterally with animals gently hand-held while the microinjector was placed into the guide cannula. Each microinjection was given at a volume of 0.5 μl/side over one minute using an infusion pump mounted with a Hamilton syringe attached to a polyethylene tubing. Animals were submitted to restraint stress 5 min after finishing microinjections. The restraint stress session consisted of confining rats individually in a restraining device, which was placed into the central corridor of the CPP apparatus. The duration of the stress session was chosen according to previous results^6^ and varied depending on the experiment as follows: 30 min (S30), a reinstating stress session for the AM251 experiment, or 15 min (S15), a non-reinstating stress session for the ACEA experiment. The ‘non-stress’ group of rats was kept in their home cages during the same time period. Immediately after finishing the restraint, all animals were tested for reinstatement (RT). More details of microinfusion procedure and restraint stress can be found in Supplementary Materials and Methods.

### Experiment 3 and 4: effects of AM251 and ACEA in the NAcore on extracellular glutamate levels after exposure to restraint stress

*Microdialysis probes* were constructed in our laboratory (see Supplementary Materials and Methods for more details) containing inlet and outlet ports and a semipermeable membrane (AN69HF, Hospal-Gambro, Meyzieu, France) with 2.0 mm of active dialyzing area at the end of the probe circuit. For simultaneous local microinjection of CB1R ligands into the NAcore, a guide cannula was attached to the microdialysis probe.

#### Surgery

The stereotaxic surgery to implant the microdialysis probe into the NAcore was performed the day after the ET and animals were allowed 18 h to recover. The procedure is described in Supplementary Materials and Methods. In brief, microdialysis probes were implanted unilaterally with the membrane placed into NAcore. Microinfusion and membrane placements were confirmed after finishing experiments (approximate locations are presented in each figure).

#### Experimental design

After animals reached the extinction criterion, they were divided into groups to observe the effects of AM251 and ACEA intra-NAcore microinjection on extracellular glutamate levels after restraint stress. For experiment 3, animals were divided into four groups to determine the effects of 5 nmol/0.5 μl/side of AM251 or vehicle (0.5 μl/side of 10% DMSO), received 5 min prior to 30 min of restraint stress (or no stress). For experiment 4, animals received 0.01 fmol/0.5 μl/side of ACEA or 0.5 μl/side of 0.1% DMSO prior to 15 min of restraint stress (or no stress). Experimental groups are summarized in Supplementary table S2.

#### Microinfusion and in vivo microdialysis procedures

On the microdialysis day, i.e. the day after surgery, animals were moved the experimental room (the same location where the CPP training was carried out) and were kept individually in their home cages for the baseline determination. Microdialysis probes were connected with FEP Teflon microdialysis tubing and perfused continuously with Ringer’s solution at a constant flow rate of 1.5 µl/min. Glutamate dialysate samples were automatically collected every 15 min in freely-moving animals. After 120 min of collecting basal samples, local microinjections were performed with animals gently hand-held while the microinjector was placed into the guide cannula. Each microinjection was given at a volume of 0.5 μl/side over one minute using an infusion pump mounted with a Hamilton syringe attached to a polyethylene tubing. Animals were submitted to restraint stress 5 min after finishing microinjection. The restraint stress session consisted of confining rats individually in a restraining device, which was placed into the central corridor of the CPP apparatus. The ‘non-stress’ group of rats was kept in their home cages during the same time period. Immediately after finishing stress, rats were reconnected to the dialysis perfusion system and sample collection continued for 1 h while animals were re-exposed to the extinguished cocaine-paired context of the CPP apparatus. More details of microdialysis procedure can be found in Supplementary Materials and Methods.

### Experiment 5: determination of context-specific changes of extracellular glutamate in the NAcore throughout the restraint stress-induced reinstatement of cocaine-CPP procedure

Microdialysis probes were constructed as described above.

Surgery to implant the microdialysis probe into the NAcore was performed as described above, the day after each CPP test, according to the experiment. Animals were allowed 18 h to recover from surgery and then submitted to in vivo microdialysis (see timelines in figures).

#### Experimental design

In order to test that expression, extinction and reinstatement of cocaine-CPP correlates with context-dependent changes in accumbal glutamate, animals were divided into 4 groups to observe the effect of re-exposure to either compartment of the CPP apparatus after completion of each phase of the CPP protocol. The first group was evaluated after the BT, i.e. animals not submitted to conditioning, and during the microdialysis procedure was re-exposed to the most preferred context according to preference scores obtained from BT. The second group was evaluated after the CT, i.e. animals submitted to conditioning, and was divided in two subgroups to observe changes in glutamate in response to re-exposure to the cocaine-paired and cocaine-unpaired CPP context. A third group was re-exposed to the cocaine-paired context during the microdialysis procedure, but after completion of the extinction phase. A fourth group, different to others, was submitted to 30 min of restraint stress before re-exposure to the cocaine-paired and cocaine-unpaired CPP context to collect microdialysis samples. Experimental groups are summarized in Supplementary table S3.

#### Microdialysis procedure

On the microdialysis day, i.e. the day after surgery, animals were moved to the experimental room (the same location where the CPP training was carried out) and were kept individually in their home cages for baseline determination. Animals were connected to the dialysis perfusion system (as described above) and membranes were perfused continuously with Ringer’s solution at a constant flow rate of 1.5 µl/min. Glutamate dialysate samples were automatically collected every 15 min in freely-moving animals. After 120 min of collecting basal samples, rats were transferred to the appropriate compartment of the CPP apparatus, i.e. the cocaine-paired or unpaired context depending on the experiment. The objective was to determine the context-dependent changes in extracellular glutamate levels within NAcore during the re-exposure to the CPP apparatus, which were compared with the baseline levels. To carry out this, the dialysate samples were collected inside the compartment for one hour.

### Experiment 6: determination of basal levels of extracellular glutamate in the NAcore following perfusion of ACEA by reverse microdialysis

Microdialysis probes were constructed as described above and surgery to implant the microdialysis probe into the NAcore was performed the day after ET.

#### Experimental design

This experiment was performed in animals that had previously attained the extinction criterion for cocaine-CPP, while rats remained in their home cages inside the experimental room without any contact with the CPP apparatus or any other stimulus. Increasing doses of ACEA [0, 10, 100 and 1000 mM] were administered by reverse microdialysis through the probe implanted in NAcore.

#### Reverse microdialysis

The sample collection procedure was carried out similarly to that described above. Firstly, baseline was determined by collecting dialysates every 15 min for 2 h by perfusing the probe implanted in NAcore with Ringer’s solution (1.5 µl/min). Then, without disconnecting the circuit, the CB1R agonist ACEA, dissolved in Ringer’s solution in a concentration of 10 mM, was perfused through the dialysis probe for one hour. Consecutively, two additional concentrations of ACEA (100 and 1000 mM) were administered in the same way. Thus, four 15-min microdialysis samples were obtained at each concentration of ACEA. With the reverse microdialysis technique, ACEA was continuously perfused into NAcore and the successive changes in extracellular glutamate levels induced by the CB1R activation could be calculated as a percentage of the baseline mean.

### Glutamate determination

Glutamate content of the perfusate was assayed by reverse-phase HPLC (Column Gemini N 3 μm; 150 × 4.6 mm; Phenomenex, USA) coupled with electrochemical detection (ESA Coulochem III; more details in Supplementary Materials and Methods, based on a previous work from our lab^38^). Glutamate was quantified by using the PC integration software EZChrom Elite v4.0 (an ESA Chromatography Data System). Changes in accumbal extracellular glutamate levels were reported as the percentage from baseline (% baseline) with the baseline being defined as the average concentration of the four last samples before exposure to the different treatments.

### Statistical analysis

Statistical analyses were performed using Prism 8.0 (GraphPad Software, La Jolla, CA, https://www.graphpad.com/) or STATISTICA 7.0 (StatSoft, United States, http://www.statsoft.com/). Statistical significance for CPP and the microdialysis data, set at p < 0.05, was determined by repeated measures (RM) one-, two- or three-way ANOVAs with post hoc multiple comparisons using the Bonferroni’s test. Depending on the experiment, Treatment (doses of CB1R ligands/vehicle), Stress (NS/S15/S30) and Context (paired/unpaired) were considered as between-subject factors, Time (15 min samples) or Test (ET vs. RT) as within-subject factors, and preference scores (Context COC − Context SAL) or glutamate levels (% baseline) as dependent variables. When only two groups were compared, statistical significance was determined by the two-tailed paired Student's *t *test. All group sizes, specific tests applied, statistical effects and statistical significance for each experiment are reported in figure legends and text in the Results section.

## Results

### Experiments 1 and 2: effects of CB1R antagonism and agonism in the NA on restraint stress-induced reinstatement of cocaine-CPP

#### Experiment 1: AM251 in the NAcore, but not NAshell, suppressed restraint stress-induced reinstatement of extinguished cocaine-CPP

**Figure 1:**
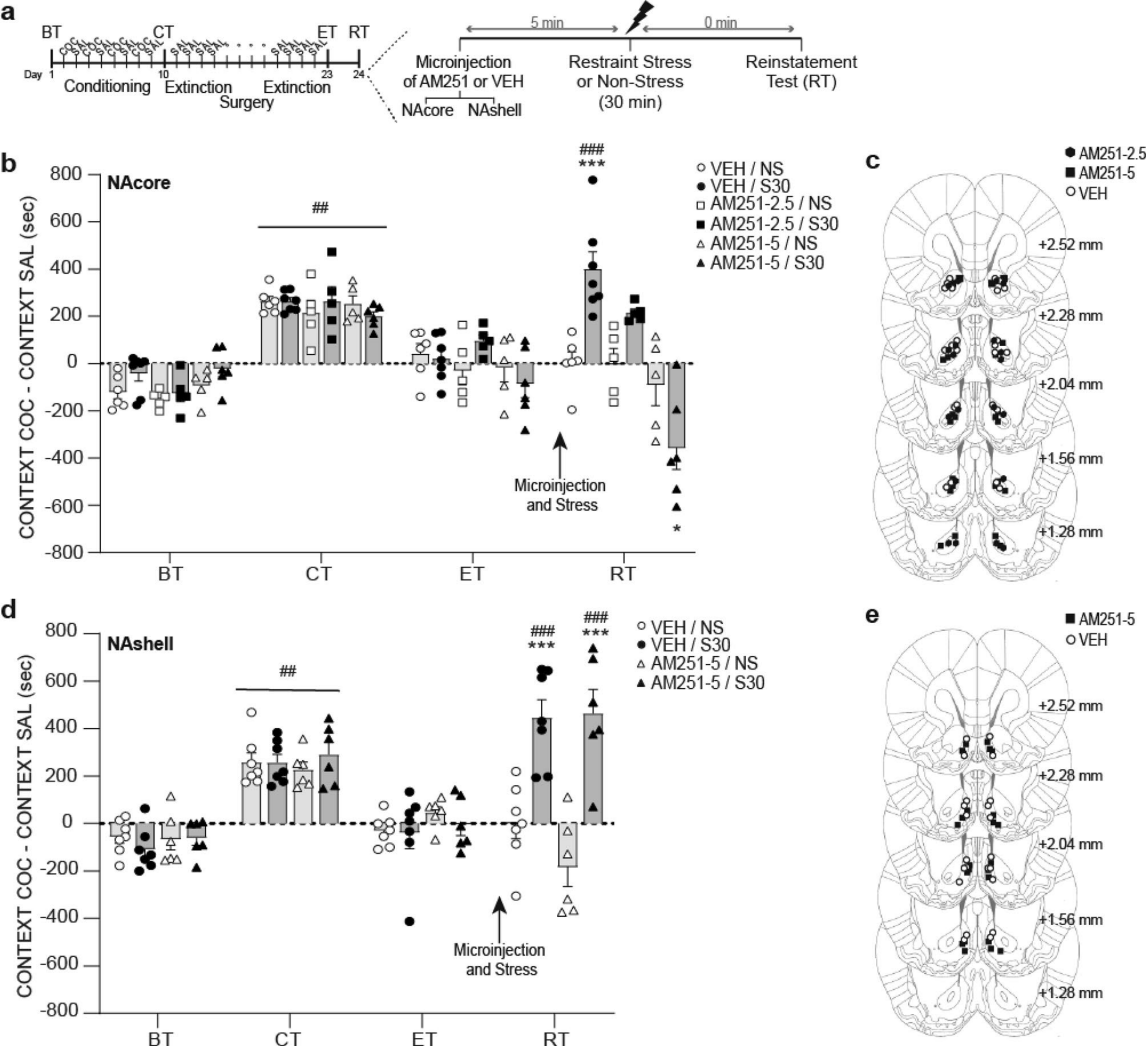
Stress-induced reinstatement of cocaine-CPP was blocked by CB1R antagonism in NAcore, but not NAshell. (**a**) Timeline of CPP procedure. Animals that met the extinction criterion received the intra-Nacore or intra-NAshell microinjection of vehicle (VEH) or a dose of AM251 (2.5 or 5 nmol/0.5 μl/side, referred as AM251-2.5 or AM251-5) 5 min before the exposure to the restraint stress for 30 min (S30), or remained in their home cages during the same time period (non-stress, NS). (**b**,**d**) Difference in preference score measured in the cocaine- and the saline-associated context during basal (BT), conditioning (CT), extinction (ET) and reinstatement (RT) tests, using CPP LIADE software, version 1.2 (http://www.liade.inv.efn.uncor.edu/) During the CT, no difference in preference score between groups was found within each experiment (^##^p < 0.001 compared to BT). Bars in dot plots represent mean ± s.e.m., n = 5–7 in each group. (**b**) Subjects microinjected with VEH into NAcore before restraint stress showed a significant increase in time spent in the cocaine-associated context during RT (^###^p < 0.001 compared to ET and ***p < 0.001 comparing with NS groups and AM251-5/S30 group in RT, Bonferroni’s multiple comparisons post-hoc test). Intra-NAcore administration of the highest dose of AM251 before restraint stress prevented reinstatement of cocaine-CPP (*p < 0.05 comparing with VEH and AM251-2.5 groups in RT, using a Bonferroni’s multiple comparisons post-hoc test). (**d**) Stress-induced reinstatement of cocaine-CPP was not prevented by the intra-NAshell administration of AM251 (***p < 0.001 compared with NS groups in RT; ^###^p < 0.001 compared with ET, Bonferroni’s multiple comparisons post-hoc test). (**c**,**e**) Schematic coronal sections of the rat brain, adapted from the stereotaxic atlas of Paxinos and Watson (2007), showing approximate bilateral locations of the microinjector tips in the NAcore (**c**) and NAshell (**e**) regions of rats included in the data analyses.

On the reinstatement day (see Fig. 1a for timeline), intra-NAcore administration of the CB1R antagonist AM251, at the highest dose (5 nmol/0.5 μl/side), was able to prevent the reinstatement of cocaine-CPP induced by 30 min of restraint stress [Fig. 1b, three-way ANOVA with RM over tests (ET vs RT): interaction test × treatment × stress F_(2,28)_ = 6.62, p < 0.05; test × treatment F_(2,28)_ = 8.58, p < 0.05; stress × treatment F_(2,28)_ = 11.19, p < 0.05]. This effect was compared with the group of animals that received an accumbal microinjection of vehicle solution before undergoing stress (VEH/S30), which is the only group that showed a significant increase in preference score in RT compared to its values in ET (p < 0.001). Although there was no significant difference between the VEH/S30 group and the group that received the lowest dose of AM251 (2.5 nmol/0.5 μl/side), there was a trend toward a reduction in preference score since no difference was found between its preference scores in ET and RT. On the other hand, Fig. 1d reveals that intra-NAshell administration of AM251 was not capable of preventing restraint stress-induced reinstatement of cocaine-CPP in animals that had previously extinguished this learning [three-way RM ANOVA: main effect for stress F_(1,22)_ = 27.57, p < 0.05; test F_(1,22)_ = 19,63, p < 0.05; interaction stress × test F_(1,22)_ = 46.08, p < 0.05]. Post-hoc analysis showed that both groups submitted to restraint stress showed a significant increase in preference score in RT when compared to ET (p < 0.001). For both experiments, groups of animals submitted to the same treatments, but not exposed to restraint stress (non-stressed group, NS), did not demonstrate reinstatement of cocaine-CPP on comparisons with the preference score measured previously in the extinction test. Figure 1c,e shows the location of AM251 microinjection cannula tips in NAcore and NAshell. Preference scores for the corresponding basal and conditioning tests are presented in the figures, showing acquisition of CPP and no differences between groups within each experiment [Fig. 1b, three-way ANOVA with RM over tests (BT vs CT): main effect for test F_(1,28)_ = 343.2, p < 0.0001; Fig. 1d, main effect for test F_(1,22)_ = 171.5, p < 0.0001].

#### Experiment 2: ACEA in the NAcore, but not NAshell, facilitated reinstatement of extinguished cocaine-CPP after exposure to a non-reinstating restraint stress session

**Figure 2:**
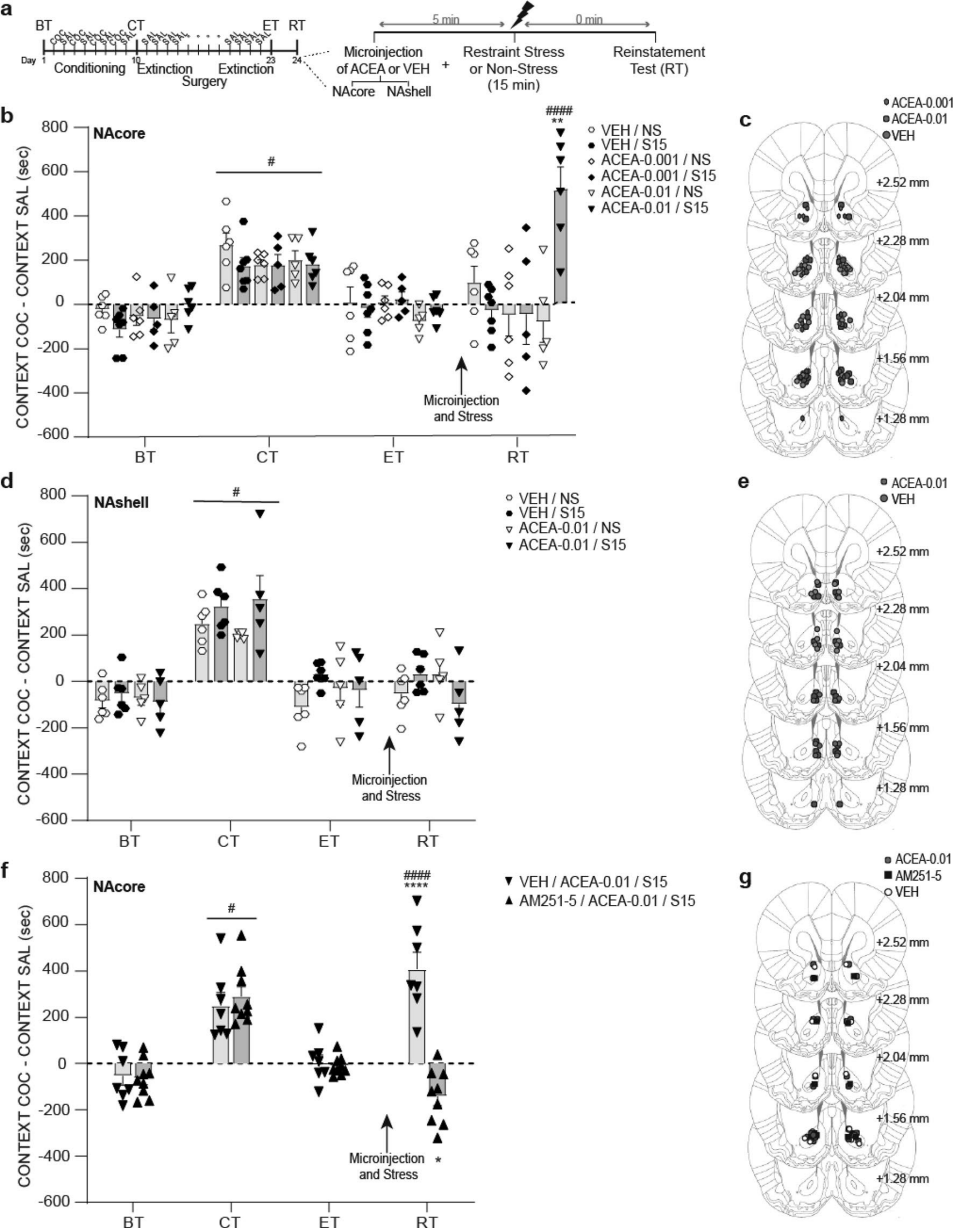
CB1R agonism within NAcore, but not NAshell, in combination with subthreshold restraint stress facilitated reinstatement of cocaine-CPP. (**a**) Timeline of CPP procedure. Animals that met the extinction criterion received the intra-Nacore or intra-NAshell microinjection of vehicle (VEH) or a dose of ACEA (0.001 or 0.01 fmol/0.5 μl/side, referred as ACEA-0.001 or ACEA-0.01) 5 min before the exposure to restraint stress for 15 min (S15) or remained in their home cages during the same time period (non-stress, NS). (**b**,**d**,**f**) Difference in preference score measured in the cocaine- and the saline-associated context during basal (BT), conditioning (CT), extinction (ET) and reinstatement (RT) tests, using CPP LIADE software, version 1.2 (http://www.liade.inv.efn.uncor.edu/). During the CT, no difference in preference score between groups was found within each experiment (^#^p < 0.05 compared to BT). Bars in dot plots represent mean ± s.e.m., n = 5–9 in each group. (**b**) Subjects microinjected with VEH or the lowest dose of ACEA (ACEA-0.001) into NAcore before subthreshold restraint stress did not lead to a significant increase in the time spent in the cocaine-associated context during RT. Intra-NAcore administration of the highest dose of ACEA (ACEA-0.01) before 15 min of restraint stress facilitated reinstatement of cocaine-CPP (**p < 0.01 comparing with the values of all the remaining groups, and ^####^p < 0.0001 comparing to ET, using Bonferroni’s multiple comparisons post-hoc test). (**d**) Reinstatement of cocaine-CPP was not facilitated by the intra-NAshell administration of ACEA before restraint stress. (**f**) Intra-NAcore pretreatment with the highest dose of AM251 prevented the facilitating effect of ACEA to induce reinstatement of cocaine-CPP (****p < 0.0001 comparing with the VEH group in RT, ^####^p < 0.0001 and *p < 0.05 compared to ET, using a Bonferroni’s multiple comparisons post-hoc test). (**c**,**e**,**g**) Schematic coronal sections of the rat brain, adapted from the stereotaxic atlas of Paxinos and Watson (2007), showing approximate bilateral locations of the microinjector tips in the NAcore (**c**,**g**) and NAshell (**d**) regions of rats included in the data analyses.

The day after ET (see Fig. 2a for timeline), intra-NAcore microinjection of the CB1R agonist ACEA at the highest dose (0.01 fmol/0.5 μl/side), before a non-reinstating restraint stress session of 15 min, induced the reinstatement of cocaine-CPP [Fig. 2b, three-way ANOVA with RM over tests (ET vs RT): interaction test × treatment F_(2,29)_ = 6.67, p < 0.05; interaction stress × treatment F_(2,29)_ = 7.60, p < 0.05; interaction test × stress × treatment F_(2,29)_ = 7.23, p < 0.05]. Post-hoc analysis showed that only this group showed a significant increase in preference score in RT compared to its values in ET (p < 0.0001). On the contrary, intra-NAshell administration of the highest dose of ACEA was not capable of facilitating reinstatement after exposure to the non-reinstating restraint stress session [Fig. 2d, three-way RM ANOVA: interaction test × stress × treatment F_(1,18)_ = 0.19; p = 0.664], showing similar results to that observed in ET and in RT for animals that were microinjected with vehicle as well as that of the non-stressed group. The pharmacological specificity of ACEA for CB1R was confirmed as we demonstrated that the prior microinfusion of AM251 in the NAcore blocked the facilitating effects of ACEA mentioned above [Fig. 2d, two-way RM ANOVA, main effect for test F_(1,14)_ = 11,90, p < 0.05; pretreatment F_(1,14)_ = 39,93, p < 0.05; and interaction test × pretreatment F_(1,14)_ = 46,16, p < 0.05, post-hoc analysis revealed a significant difference between groups pretreated with VEH or AM251 in the reinstatement test, p < 0.0001]. Figure 2c,e,g shows the location of ACEA, and AM251, microinjection cannula tips in the NAcore and NAshell. Preference scores for the corresponding basal and conditioning tests are presented in figures, showing acquisition of CPP and no differences between groups within each experiment [Fig. 2b, three-way ANOVA with RM over tests (BT vs CT): main effect for test F_(1,29)_ = 113.3, p < 0.0001; Fig. 2d, three-way RM ANOVA: main effect for test F_(1,18)_ = 113.2, p < 0.0001; Fig. 2f, two-way RM ANOVA: main effect for test F_(1,14)_ = 59.23, p < 0.0001].

### Experiments 3 and 4: effects of CB1R antagonism and agonism in the NAcore on extracellular glutamate levels after exposure to restraint stress

#### Experiment 3: AM251 in NAcore suppressed the increase of extracellular glutamate during re-exposure to the cocaine-paired context after a reinstating restraint stress session

**Figure 3:**
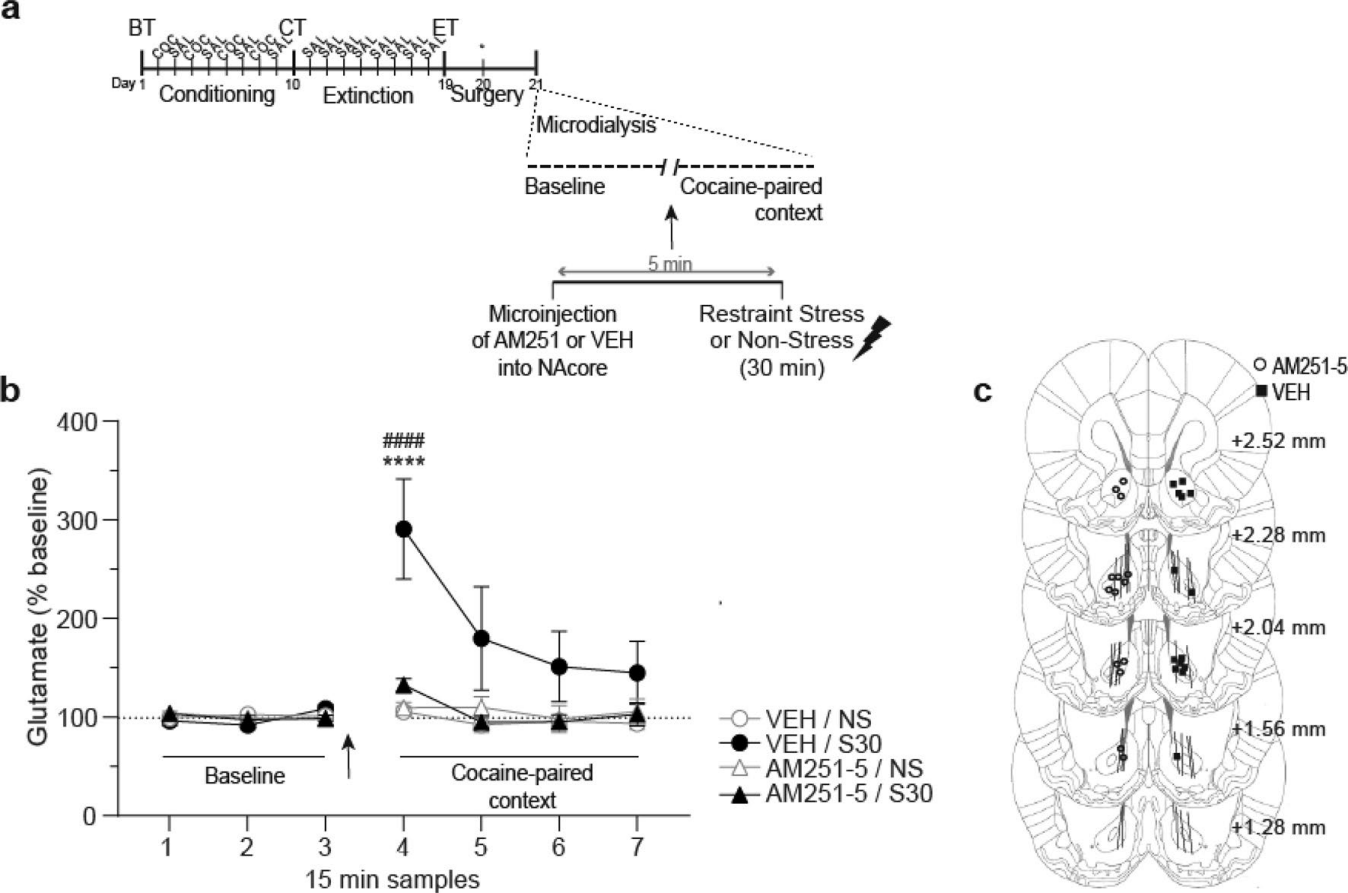
Stress-induced increase of extracellular glutamate was blocked by CB1R antagonism in NAcore. (**a**) Timeline of CPP procedure. Stereotaxic surgery for microdialysis probe implantation was performed once animals reached extinction criteria. The day after surgery, in vivo microdialysis was used to sample (every 15 min) extracellular glutamate in the NAcore both before (baseline determination, in home cage) and after AM251 (or VEH) intra-NAcore administration and 30 min of restraint stress (or non-stress). Following these treatments, samples were collected inside the cocaine-paired compartment of the CPP apparatus. Glutamate was quantified using EZChrom Elite software, version 4.0 (http://www.agilent.com). (**b**) AM251 local pretreatment attenuated the restraint stress-induced increase in glutamate of NAcore measured in the cocaine-paired context (****p < 0.0001 compared with other groups at the same time point; ^####^p < 0.0001 compared with the last baseline sample, using a Bonferroni’s post-hoc test). Values represent percentage of increase in glutamate release from baseline (mean ± s.e.m.) for each 15 min sample, n = 7 for each group. Arrow indicates the microinjection and restraint stress treatment. (**c**) Approximate unilateral locations of microinfusions of AM251 performed into NAcore together with the dialysis membranes placements (solid lines) of rats implanted with the dual microinjection-microdialysis probes. Numbers to the right indicate millimeters from the Bregma. Symbols represent the different microinjections administered.

Interestingly, in animals that previously extinguished cocaine-CPP (see Fig. 3a for timeline), we found that the microinjection of AM251 (5 nmol/0.5 μl/side) directly into NAcore, before the restraint stress session of 30 min (AM251-5/S30), suppressed the significant increase in extracellular glutamate levels observed in the vehicle group (VEH/S30), in the first sample collected inside the cocaine-paired context after stress [Fig. 3b, three-way ANOVA with RM over time (15 min samples) revealed a significant effect for stress F_(1,24)_ = 5.51, p < 0.05; time F_(6,144)_ = 8.69, p < 0.05; interaction time × stress F_(6,144)_ = 6.76, p < 0.05; interaction treatment × stress F_(1,24)_ = 5.46, p < 0.05; and interaction treatment × stress × time F_(6,144)_ = 4.68, p < 0.05]. Post-hoc analysis showed a significant increase in glutamate for the VEH/S30 group compared to samples collected during the baseline determination (p < 0.0001) and to the first sample collected inside the cocaine-paired context for the other groups (p < 0.0001). No changes in glutamate levels were found in non-stressed animals or in those microinjected with vehicle or AM251. Locations of the AM251 microinjection and the active membrane of the microdialysis probe are presented in Fig. 3c.

#### Experiment 4: ACEA in the NAcore facilitated the increase of extracellular glutamate levels during re-exposure to the cocaine-paired context after a non-reinstating restraint stress session

**Figure 4:**
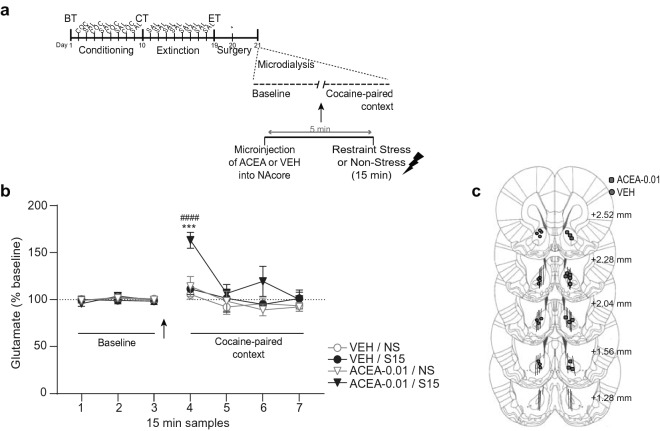
CB1R agonism within NAcore, in combination with subthreshold restraint stress, induced an increase in extracellular glutamate in NAcore. (**a**) Timeline of CPP procedure. Stereotaxic surgery for microdialysis probe implantation was performed once animals reached extinction criteria. The day after surgery, in vivo microdialysis was used to sample (every 15 min) extracellular glutamate in NAcore both before (baseline determination, in home cage) and after ACEA (or VEH) intra-NAcore administration and 15 min of restraint stress (or non-stress). Following treatments, samples were collected inside the cocaine-paired compartment of the CPP apparatus. Glutamate was quantified using EZChrom Elite software, version 4.0 (http://www.agilent.com). (**b**) ACEA local pretreatment potentiated the increase in glutamate of NAcore measured in the cocaine-paired context immediately after a subthreshold session of restraint stress (***p < 0.001 compared with other groups at the same time point; ^####^p < 0.0001 compared with the last baseline sample, Bonferroni’s multiple comparisons post-hoc test). Values represent percentage of increase in glutamate release from baseline (mean ± s.e.m.) for each 15 min sample, n = 6–7 for each group. Arrow indicates the microinjection and restraint stress treatment. (**c**) Approximate unilateral locations of microinfusions of ACEA performed into NAcore together with the dialysis membranes placements (solid lines) of rats implanted with the dual microinjection-microdialysis probes. Numbers to the right indicate millimeters from the Bregma. Symbols represent the different microinjections administered.

Once the extinction of cocaine-CPP was confirmed (see Fig. 4a for timeline), microdialysis experiments demonstrated that local microinfusion of ACEA (0.01 fmol/0.5 μl/side) into the NAcore, before the subthreshold restraint stress session, resulted in increased extracellular glutamate levels during the first 15 min of permanence inside the cocaine-paired context, compared with animals microinjected with vehicle or the ‘ACEA/NS’ group [Fig. 4b, three-way ANOVA with RM over time: interaction time × treatment × stress F_(6,132)_ = 2.23, p < 0.05; interaction time × treatment F_(6,132)_ = 3.31, p < 0.05]. Post-hoc analysis showed a significant increase in glutamate for the ACEA-0.01/S15 group compared to samples collected during the baseline determination (p < 0.0001) and to the first sample collected inside the cocaine-paired context for the other groups (p < 0.001). Locations of the ACEA microinjection and the active membrane of the microdialysis probes are presented in Fig. 4c.

### Experiment 5: context specific changes in extracellular glutamate in the NAcore throughout the restraint stress-induced reinstatement of cocaine-CPP procedure

**Figure 5:**
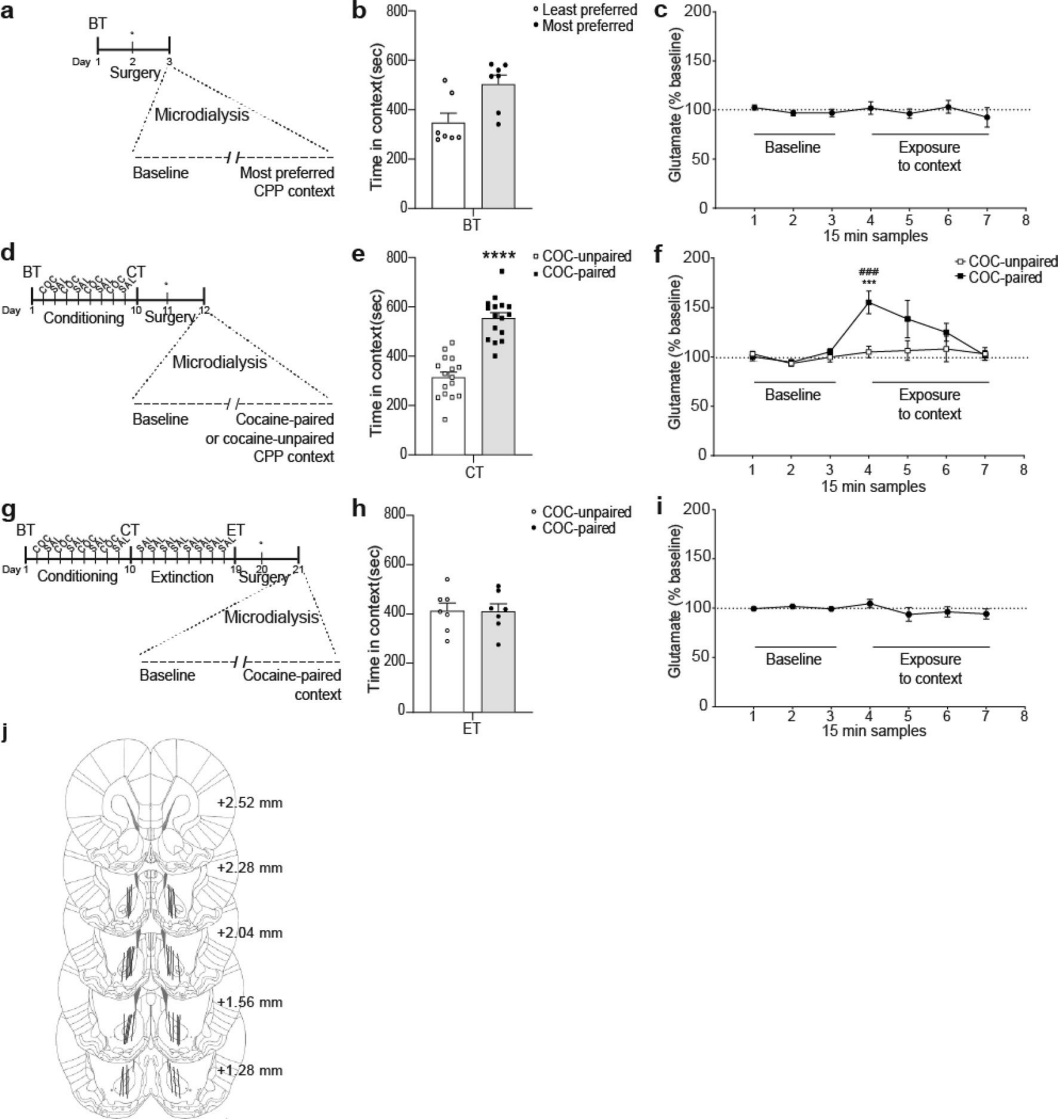
Enhancement of extracellular glutamate in NAcore depended on the CPP context and on the phase of the reinstatement of cocaine-CPP procedure. (**a**,**d**,**g**) Timelines of CPP procedures. The day after surgery for probe implantation, in vivo microdialysis was performed to sample (every 15 min) extracellular glutamate in NAcore both before (baseline, in home cage) and after transfer of animals to either compartment of the CPP apparatus. (**b**,**e**,**h**) Time spent (seconds) in each CPP context during the previous corresponding test, using CPP LIADE software, version 1.2 (http://www.liade.inv.efn.uncor.edu/). Bars in dot plots represent mean ± s.e.m. The two-tailed paired Student’s *t* test was used to analyze these data. (**c**,**f**,**i**) For microdialysis, values represent percentage of increase in glutamate release from baseline (mean ± s.e.m.) for each sample using Bonferroni’s multiple comparisons post-hoc test was used. Glutamate was quantified using EZChrom Elite software, version 4.0 (http://www.agilent.com). Naïve animals (n = 7) that showed a statistically non-significant trend to unconditioned preference for either CPP compartment during BT (**b**) did not exhibit any change in extracellular glutamate when they were transferred to the most-preferred context (**c**). In contrast, after the conditioning phase, animals that expressed a significant conditioned preference for the cocaine-paired context during CT (**e**, ****p < 0.001, n = 16**)** then demonstrated a context-specific augmentation of extracellular glutamate (**f**, ***p < 0.001 compared with the cocaine-unpaired context group at the same time point; ^###^p < 0.001 compared with the last baseline sample, n = 8 for each group). After confirming extinction of the cocaine-CPP behavior (**h**), animals did not exhibit any significant change in extracellular glutamate when they were re-exposed to the previous cocaine-paired context (**i**, n = 7). (**j**) Summary illustration of the locations of the active membrane (solid lines) of the microdialysis probes in the NAcore of subjects that were included in the analysis of the data presented in this figure. Numbers to the right indicate millimeters from the Bregma.

**Figure 6:**
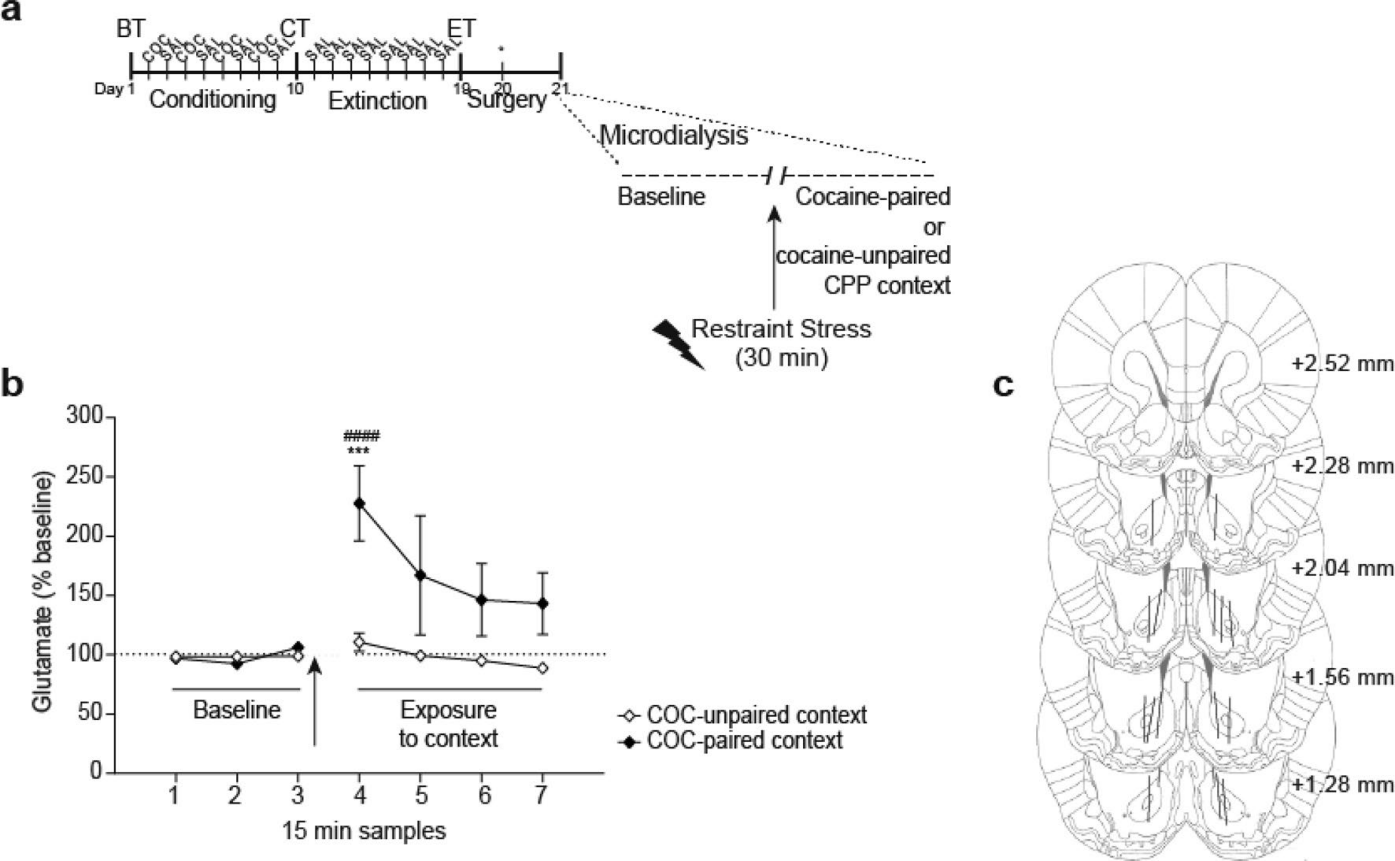
Restraint stress caused an enhancement of extracellular glutamate in NAcore that depended on the CPP context. (**a**) Timeline of CPP procedure. After confirming extinction of the cocaine-CPP behavior (data not shown, no differences in preferences scores between groups, Student’s *t* test, cocaine-paired vs unpaired context: t = 1.08, p = 0.297), animals were submitted to probe implantation the day before collecting samples. In vivo microdialysis was performed to sample (every 15 min) extracellular glutamate in NAcore both before (baseline, in home cage) and after transfer of animals to either compartment of the CPP apparatus. After 120 min of collecting basal samples inside the home cage, animals were submitted to restraint stress for 30 min and then re-exposure to the previous cocaine-paired or -unpaired context. Glutamate was quantified using EZChrom Elite software, version 4.0 (http://www.agilent.com). (**b**) A significant increase of glutamate in NAcore was observed when animals were re-exposed to the cocaine-paired, but not the cocaine-unpaired context, after being exposed to restraint stress (***p < 0.001 compared with cocaine-unpaired context group at the same time point; ^####^p < 0.0001 compared with the last baseline sample, Bonferroni’s multiple comparisons post-hoc test). Values represent percentage of increase in glutamate release from baseline (mean ± s.e.m.) for each 15 min sample, n = 7–8 for each group. Arrow indicates the restraint stress exposure. (**c**) Unilateral locations of microdialysis membranes (solid lines) in NAcore for subjects that were included in the present analysis. Numbers to the right indicate millimeters from the Bregma.

To test whether the increase in accumbal glutamate following restraint stress occurred in response to the cocaine-paired context and was not due to either the stress or/and the CPP apparatus per se or to the experimental room (unspecific cues), we corroborated the influence of the cocaine-paired context at different stages of the cocaine-CPP behavioral procedure. Locations of the active membrane of the probes, for the four following experiments, are presented in Figs. 5j and 6d.

#### Experiment 5A: before conditioning, re-exposure to the most preferred context did not induce enhancement of extracellular glutamate in the NAcore

For this experiment, we used animals that were not submitted to cocaine conditioning (see Fig. 5a for timeline) and did not show an unconditioned preference for either CPP compartment [Fig. 5b, Student’s *t* test, least- vs most-preferred context: t = 2.08, p = 0.083]. Figure 5c reveals that there were no significant differences [one-way RM ANOVA: no effect of time F_(6,36)_ = 0.61, p = 0.716] in extracellular glutamate levels between samples collected during the baseline determination and samples collected inside the CPP context that was slightly but not statistically more preferred during the previous BT.

#### Experiment 5B: after conditioning, re-exposure to the cocaine-paired, but not to the cocaine-unpaired, context induced enhancement of extracellular glutamate in the NAcore

Here, we demonstrated that there was a significant rise in the extracellular glutamate levels within the NAcore during the first 15 min of re-exposure to the cocaine-paired context, but not to the cocaine-unpaired context (the saline-paired context) [Fig. 5f, two-way RM ANOVA: main effect for time F_(6,90)_ = 5.33, p < 0.0001; context F_(1,15)_ = 5.42, p < 0.05; interaction time × context F_(6,90)_ = 3.08, p < 0.05], in animals that had previously shown a significant preference for this compartment during the conditioning test [see Fig. 5d for timeline and Fig. 5e for behavioral results, Student’s *t *test, cocaine-paired vs unpaired context: t = 5.92, p < 0.0001]. Post-hoc analysis revealed a significant increase in glutamate for the cocaine-paired group compared to samples collected during baseline determination (p < 0.001) and to the first sample collected inside the cocaine-paired context for the saline-paired group (p < 0.001).

#### Experiment 5C: after extinction, re-exposure to the cocaine-paired context did not induce enhancement of extracellular glutamate in the NAcore

After confirming extinction of cocaine-CPP [see Fig. 5g for timeline and Fig. 5h for behavioral results, Student’s *t* test, cocaine-paired vs unpaired context: t = 0.02, p = 0.984], we did not find any significant changes in the extracellular glutamate levels within NAcore, during the re-exposure to the previously extinguished cocaine-paired context [Fig. 5i, one-way RM ANOVA: no effect of time F_(6,42)_ = 1.14, p = 0.353].

#### Experiment 5D: context-specific enhancement of extracellular glutamate in the NAcore occurred after restraint stress in rats that extinguished cocaine-CPP

For animals that had previously attained the extinction criterion for cocaine-CPP (see Fig. 6a for timeline), we have demonstrated that 30 min of restraint stress and immediate re-exposure to the cocaine-paired context induced a significant increase in accumbal glutamate in the first collected sample, compared with animals that were re-exposed to the cocaine-unpaired context [Fig. 6b, two-way RM ANOVA: main effect for time F_(6,72)_ = 5.67, p < 0.05; context F_(1,12)_ = 4.80, p < 0.05; interaction time × context F_(6,72)_ = 4.21, p < 0.05; post-hoc analysis showed a significant difference between groups at the same time point, p < 0.001, and a significant increase in glutamate compared to the last baseline sample, p < 0.0001].

### Experiment 6: basal levels of accumbal extracellular glutamate were regulated by the activation of CB1R

**Figure 7:**
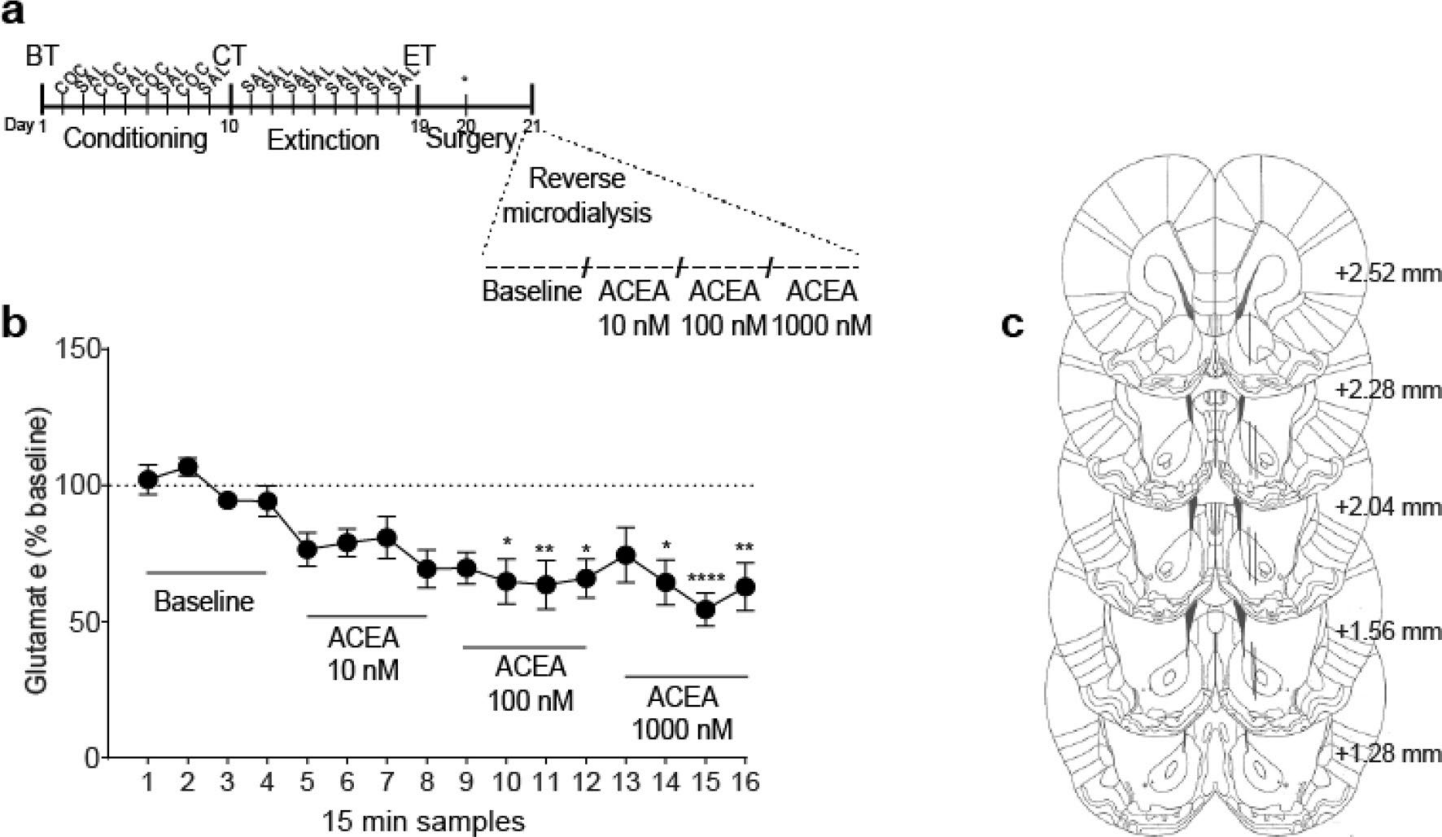
Effect of the CB1R agonist ACEA on basal extracellular glutamate levels in NAcore. (**a**) Timeline for CPP procedure. Animals that reached the extinction criteria were selected to receive increasing and consecutive doses of ACEA by reverse microdialysis [0, 10, 100 and 1000 mM]. Following the baseline, four samples for each concentration were collected. (**b**) Intra-NAcore local perfusion of ACEA significantly decreased extracellular glutamate in a concentration-dependent manner (*p < 0.05, **p < 0.01 and ****p < 0.0001 compared with baseline, using Bonferroni’s multiple comparisons post-hoc test, n = 7). Glutamate was quantified using EZChrom Elite software, version 4.0 (http://www.agilent.com). (**c**) Locations of microdialysis membranes (solid lines) in NAcore for subjects that were included in the present analysis. Numbers to the right indicate millimeters from the Bregma.

Increasing doses of ACEA [0, 10, 100 and 1000 mM] administered by reverse microdialysis through the probe implanted in the NAcore caused a reduction in the basal levels of extracellular glutamate in a dose-dependent manner [Fig. 7b, one-way RM ANOVA, main effect for time F_(15,90)_ = 9.11, p < 0.0001; post-hoc analysis showed significant differences with samples collected during baseline determination, p < 0.05]. This experiment was performed in animals that had previously attained the extinction criterion for cocaine-CPP (see Fig. 7a for timeline) and inside the experimental room, while the rats remained in their home cages. Figure 7c shows the locations of the active membrane of the microdialysis probes.

## Discussion

Our main findings showed that antagonism of CB1R by AM251 in the NAcore, but not NAshell, dose-dependently prevented restraint stress-induced reinstatement of cocaine-CPP, while agonism of CB1R by ACEA potentiated reinstatement after a subthreshold restraint stress session. Interestingly, this suppressive or facilitatory influence of AM251 or ACEA, respectively, was mirrored by a suppression or a potentiation of the cocaine-paired context-specific increase in extracellular glutamate in the NAcore following restraint stress.

### CB1R in the NAcore, but not NAshell, participates in stress-induced reinstatement of cocaine-CPP

In agreement with our study, two other studies, which also used the CPP paradigm and forced swimming or restraint as stressors^24,25^, demonstrated that systemic or intra-ventral tegmental area administration of AM251 prevented stress-induced reinstatement of cocaine seeking. However, some other results under the self-administration paradigm reported contrasting results. For example, systemic administration of another CB1R antagonist/inverse agonist (SR141716A^17^) or the intracerebroventricular microinjection of AM251^26^ did not block footshock-induced reinstatement of cocaine-seeking behavior, whereas the stress-potentiated (footshock plus a subthreshold dose of cocaine) reinstatement was abrogated by systemic AM251^27^. Moreover, the corticotropin-releasing factor-induced reinstatement of cocaine seeking was prevented by AM251 administration^26^. Differences may be attributed to methodological variables, such as the type of stressor and the previous history with the drug, the species/strains used, the pharmacological agents and different learning paradigms (CPP versus self-administration)^3,28^. In addition, the doses and routes of administration of CB1R ligands may determine the overall effects. In fact, CB1R is the most widely expressed presynaptic Gi/o-protein coupled receptor in the brain^15,16^ and is involved in the modulation of multiple neurobehavioral processes, suggesting that the simultaneous blockade of CB1R could elicit complex and antagonistic effects.

Besides suppression of reinstatement, a statistical non-significant trend to avoid the cocaine-paired context was observed after the highest dose of AM251 and restraint stress, but not when AM251 was administered alone (without stress). This observation may be understood as a state of anhedonia induced by stress in combination with CB1Rs blockade which leads animals to avoid the cocaine-paired context. This could be related to the ability of CB1R antagonists/inverse agonists to attenuate motivational effects of cocaine and natural rewards^19,27^, and to exacerbate stress-induced anhedonia. Although this role may be attributed to a more peripheral action^39^, the systemic administration of AM251 did not produce avoidance for the cocaine context after stress^25^. Further investigations are needed to dissociate bidirectional effects of CB1R antagonists/inverse agonists on conditioned reward and aversion.

The local pharmacological treatment used here reported the differential role for CB1R within the subregions of the NA in stress-induced reinstatement, consistent with previous work from our laboratory that revealed the involvement of glutamatergic NMDA receptors within the NAcore, but not NAshell, in our model^6^. Related to this, various studies have reported differential contributions for these subregions in reinstatement of cocaine-seeking^40–43^. While the NAshell is important in modulating the incentive-motivational value of a drug, the NAcore is responsible for initiating a reward-related motor action that underlies the expression of learned behaviors (reviewed in^29^). Although it is known that CB1R is highly expressed in the NA and modulates glutamatergic transmission in this area^31,44^, the differential contributions of CB1R between NAcore and NAshell in drug-related behaviors have not yet been elucidated. The only study to date that has explored the role of CB1R within the NA in reinstatement of cocaine self-administration showed that local administration of AM251, without discriminating between NAcore and NAshell, suppressed drug-induced reinstatement^18^.

As a complementary analysis, we have shown that a mild restraint stress session, which does not induce reinstatement by itself, in combination with intra-NAcore, but not intra-NAshell, administration of CB1R agonist ACEA, triggered reinstatement of extinguished cocaine-CPP. This suggests that CB1R activation may potentiate the effect of a subthreshold restraint stress session. Supporting this idea, it has been previously demonstrated that CB1R agonism, by administering WIN55,212-2 or HU210 alone, reinstated drug-seeking behavior for several drugs^17,45–48^ or potentiated the effects of certain stimuli that do not trigger reinstatement by themselves, such as drug-associated cues^46,49^ or subthreshold doses of an alpha-adrenergic antagonist^25^ or MDMA^50^. On another note, accumulating evidence has indicated that different types of stress or anxiety conditions can modify in vivo the endocannabinoid levels and can influence CB1R signaling within several brain regions implicated in drug-seeking behavior (see reviews^35,51^). For example, repeated restraint stress elicited an increase in 2-Arachidonylglycerol content within forebrain regions, including the NA^52^. Consistently, pharmacological manipulation of the CB1R can regulate stress-induced changes, such as the activation of the hypothalamic–pituitary–adrenal axis^53,54^, which is known to sensitize the function of reward areas through a glucocorticoid mechanism. Data presented here support the idea that the endocannabinoid system^55^ as well as stressful events^27,56^ can act as ‘occasion setters’, i.e. both may potentiate the function of drug-paired stimuli in driven behavior, probably by potentiating the sensitivity of brain reward-related pathways to these stimuli.

### CB1R in the NAcore regulates extracellular glutamate under reinstatement conditions of extinguished cocaine-CPP

Our current findings clearly showed that the intra-NAcore administration of AM251 suppressed the cocaine-paired context-specific increase of extracellular glutamate after restraint stress. Consistently, systemic AM251 prevented the glutamate increase in the NA induced by a cocaine injection, under reinstatement conditions^18^. Although this latter study did not discriminate between the NA subregions when drawing conclusions, the location of the active microdialysis membranes primarily in the NAcore suggests that the cocaine-induced increase in extracellular glutamate may occur preferentially within that subregion. Taken together, these results are in agreement with the crucial role of the glutamate transmission within the NAcore in drug-, cue- and stress-induced reinstatement^10,11,57,58^. Thus, this study supports the conclusion that a glutamate-related mechanism underlies the reinstatement-suppressing effects of AM251. Consistently, our study also showed an enhancement in extracellular glutamate in the NAcore after combining ACEA with a non-reinstating restraint stress session, which is paralleled by our behavioral results.

Interestingly, our data support the idea that CB1R can mediate the impact of exposure to not only restraint stress, but also a drug-associated environment, on glutamatergic transmission within the NAcore, in order to influence reinstatement to cocaine-seeking. Specifically, acute exposure to restraint stress in rats previously submitted to extinction of cocaine-CPP triggered accumbal glutamate release during subsequent re-exposure to the cocaine-paired context, but not to the cocaine-unpaired context. This observation shows that glutamate augmentation was context-dependent and not elicited simply by restraint stress. We hypothesize that this transient increase in glutamate efflux after stress depends on the previous drug history and may reflect a heightened anticipation of the rewarding effects obtained with the drug in a specific compartment. It should be noted that there was a close relationship demonstrated here between the expression, extinction and reinstatement of cocaine-CPP and the context-dependent changes in accumbal glutamate. In other studies that also used in vivo microdialysis, contexts or cues associated with drug intake caused the release of glutamate in the NA, in animals trained to self-administer cocaine or heroin^59–62^. In addition, we demonstrated here the suppressing effects of extinction learning on the drug-paired CPP context-induced increase in accumbal extracellular glutamate, consistently to that obtained in self-administration studies^61,63,64^. All these experiments support the hypothesis that the secondary reinforcing properties of drugs are modulated in part by glutamate in the NAcore, and thereby CB1R may interact with these inputs to influence psychostimulant-seeking behavior.

Regarding the mechanism involved, ex vivo electrophysiological studies have demonstrated that several forms of excitatory synaptic plasticity in the NA require endocannabinoid signaling^31,44,65,66^. Nevertheless, there is virtually no information about the CB1R-mediated presynaptic modulation of glutamate release in vivo after a relapse-triggering stimulus. Several interconnected mechanisms can be proposed to explain how the pharmacological manipulation of CB1R may induce, or contribute at least in part, to changes in extracellular glutamate in the NA and therefore reinstatement. Firstly, we hypothesize that the suppressing effect of AM251 on restraint stress-induced reinstatement of cocaine-CPP may be attributed to an initial increase of extracellular glutamate, which may subsequently suppress the stress-induced synaptic glutamate release and reinstatement by activation of presynaptic metabotropic glutamate receptors (mgluR2/3). Accordingly, mgluR2/3 activation by LY379268 prevented stress-induced reinstatement of cocaine seeking^7^. Moreover, this hypothesis is based on previous evidence showing that systemic administration of AM251 increases extracellular glutamate levels in the NA by itself and that the blockade of accumbal mgluR2/3 by LY341495 suppressed the antagonism of cocaine-induced reinstatement by AM251^18^. Thus, restoration of basal extracellular glutamate in the NAcore, which was seen to decrease after extinction or withdrawal from repeated cocaine^63,67,68^, suppressed the subsequent cocaine-induced glutamate release and reinstatement by restoring the inhibitory presynaptic tone of mgluR2/3^69,70^. As a counterpart to that observed with AM251, the potentiated effect of ACEA, together with a non-reinstating restraint stress session, on glutamate and reinstatement may be attributed to an initial reduction in basal levels of extracellular glutamate which may result in lower inhibition of mgluR2/3, favoring the presynaptic glutamate release triggered by restraint stress. Consistently with this hypothesis, we observed that the intra-NA local and continued perfusion of ACEA decreased basal extracellular glutamate levels in vivo, in the absence of any reinstating stimulus. Other in vivo evidence showed that i.p. administration of CB1R agonists (THC or WIN55,212-2) reduced glutamate in accumbal dialysates^71,72^. Complementary to our results, the local and continued perfusion of the CB1R antagonist SR141716A, by reverse microdialysis, increased extracellular glutamate in the NA^73^, which was also consistent with the in vivo increase in accumbal glutamate observed after i.p. administration of AM251 or SR141716A^18,73^. Together, these in vivo findings are consistent with a tonic inhibition of CB1R on glutamate release, and in this way CB1R agonism would reduce, while CB1R antagonism/inverse agonism would facilitate, presynaptic glutamate release through the canonical mechanism of action described for CB1R in accumbal slices^31,66^. Secondly, another hypothesis that relates CB1R and mgluR2/3 activities, but not directly via basal glutamate, is based on the ability of CB1R to sequester Gi/o proteins after activation^74^, which may prevent mgluR2/3 autoreceptors from signaling. However, the implication of this in vitro study on in vivo neurochemical changes or behavior has not been investigated.

A third hypothesis can be related to the activity of CB1R expressed in astrocytes^75,76^. A recent work, using NA slice electrophysiology from cocaine-experienced rats, showed that activation of CB1R causes an excitatory event that typically corresponds to astrocytic glutamate release^75^. However, this mechanism seems not to impact significantly on those in vivo changes in extracellular glutamate detected during the perfusion of ACEA in our study. In fact, the neuronal origin of basal extracellular glutamate regulated by CB1R is supported by the previous observation that administration of AM251 alone induced an increase of extracellular glutamate in NA in a TTX-dependent manner^18^. It should be noted that after chronic perfusion of methAEA, a partial agonist of CB1R with a more robust effect on astrocytic CB1R than on presynaptic CB1R, restored the impaired glutamate homeostasis in cocaine-experienced rats, and subsequently prevented reinstatement^75^. This latter mechanism can be proposed as an alternative or indirect pathway to obtain similar results to that proposed for the acute administration of AM251^18^. In both cases, the inhibition of reinstatement might be an overall consequence of stimulating mgluR2/3, via sustained astrocytic glutamate release by a partial agonist^75^ or via the facilitating effect of a potent, selective CB1R antagonist/inverse agonist on neuronal glutamate release^18^. Conversely, glutamate release from astrocytes occurs in those that are reactive^77^ apparently in response to drug-associated cues and stress^78,79^, and therefore it is likely that the context-specific elevation of extracellular glutamate after restraint stress in our study may be attributed to a summation between glutamate release from reactive astrocytes (mediated by CB1R or mgluR5) and that from presynaptic terminals. Finally, future studies should also be directed toward exploring whether or how additional actions of CB1R ligands on other receptors, such as the GABA(A), mu-opioid and the vanilloid TRPV1 receptors, contribute to regulate drug seeking and glutamate release in vivo^80–82^.

It is important to mention that although CB1R are also expressed in GABAergic terminals in NA^83^, it has been shown that AM251-induced inhibition of cocaine-triggered reinstatement does not involve changes in extracellular GABA in NA^18^. In the same study, the participation of dopaminergic transmission in the role of CB1R was also discarded, consistent with evidence reporting that CB1Rs are not expressed in dopamine terminals^15^ and do not directly modulate dopamine release in the NA^73,84^.

In conclusion, the findings presented here support the hypothesis that stress exposure and CB1R activation may interact in NAcore to promote relapse to cocaine seeking by modulating glutamate transmission. Importantly, the context-specific changes in extracellular glutamate after restraint, together with the suppressive effect of AM251, clearly demonstrated a CB1R-dependent glutamatergic mechanism in the processing of the motivational significance of drug-related memories. Our contribution to better understanding the key features of the endocannabinoid system may be useful in the development of pharmacological treatments for patients with drug abuse- and stress-related disorders.

## Supplementary Information

Supplementary Information.

## Data Availability

The data is available from the corresponding author upon request.

## References

[1] Sinha R (2001). How does stress increase risk of drug abuse and relapse?. Psychopharmacology.

[2] McCabe SE, Cranford JA, Boyd CJ (2016). Stressful events and other predictors of remission from drug dependence in the United States: Longitudinal results from a national survey. J. Subst. Abuse Treat..

[3] Mantsch JR, Baker DA, Funk D, Le AD, Shaham Y (2016). Stress-induced reinstatement of drug seeking: 20 years of progress. Neuropsychopharmacology.

[4] Torres-Berrio A, Cuesta S, Lopez-Guzman S, Nava-Mesa MO (2018). Interaction between stress and addiction: Contributions from Latin-American neuroscience. Front. Psychol..

[5] Kalivas PW, Volkow ND (2011). New medications for drug addiction hiding in glutamatergic neuroplasticity. Mol. Psychiatry.

[6] De Giovanni LN, Guzman AS, Virgolini MB, Cancela LM (2016). NMDA antagonist MK 801 in nucleus accumbens core but not shell disrupts the restraint stress-induced reinstatement of extinguished cocaine-conditioned place preference in rats. Behav. Brain Res..

[7] Martin-Fardon R, Weiss F (2012). (–)-2-oxa-4-aminobicylco[3.1.0]hexane-4,6-dicarboxylic acid (LY379268) and 3-[(2-methyl-1,3-thiazol-4-yl)ethynyl]piperidine (MTEP) similarly attenuate stress-induced reinstatement of cocaine seeking. Addict. Biol..

[8] Wang B (2005). Cocaine experience establishes control of midbrain glutamate and dopamine by corticotropin-releasing factor: A role in stress-induced relapse to drug seeking. J. Neurosci..

[9] Garcia-Keller C (2020). N-Acetylcysteine treatment during acute stress prevents stress-induced augmentation of addictive drug use and relapse. Addict. Biol..

[10] McFarland K, Davidge SB, Lapish CC, Kalivas PW (2004). Limbic and motor circuitry underlying footshock-induced reinstatement of cocaine-seeking behavior. J. Neurosci..

[11] McFarland K, Lapish CC, Kalivas PW (2003). Prefrontal glutamate release into the core of the nucleus accumbens mediates cocaine-induced reinstatement of drug-seeking behavior. J. Neurosci..

[12] Bechard AR (2021). Role of prefrontal cortex projections to the nucleus accumbens core in mediating the effects of ceftriaxone on cue-induced cocaine seeking. Addict. Biol..

[13] Popoli M, Yan Z, McEwen BS, Sanacora G (2011). The stressed synapse: The impact of stress and glucocorticoids on glutamate transmission. Nat. Rev. Neurosci..

[14] Koob GF (2014). Addiction as a stress surfeit disorder. Neuropharmacology.

[15] Herkenham M (1991). Characterization and localization of cannabinoid receptors in rat brain: A quantitative in vitro autoradiographic study. J. Neurosci..

[16] Mackie, K. Distribution of cannabinoid receptors in the central and peripheral nervous system. In *Handbook of experimental pharmacology*, 299–325. 10.1007/3-540-26573-2_10 (2005). 10.1007/3-540-26573-2_10 16596779

[17] De Vries TJ (2001). A cannabinoid mechanism in relapse to cocaine seeking. Nat. Med..

[18] Xi ZX (2006). Cannabinoid CB1 receptor antagonist AM251 inhibits cocaine-primed relapse in rats: Role of glutamate in the nucleus accumbens. J. Neurosci..

[19] Adamczyk P (2012). The effects of cannabinoid CB1, CB2 and vanilloid TRPV1 receptor antagonists on cocaine addictive behavior in rats. Brain Res..

[20] Filip M (2006). Involvement of cannabinoid CB1 receptors in drug addiction: Effects of rimonabant on behavioral responses induced by cocaine. Pharmacol. Rep..

[21] Jing L, Qiu Y, Zhang Y, Li JX (2014). Effects of the cannabinoid CB(1) receptor allosteric modulator ORG 27569 on reinstatement of cocaine- and methamphetamine-seeking behavior in rats. Drug Alcohol Depend..

[22] Yu LL (2011). Effects of cannabinoid CB(1) receptor antagonist rimonabant on acquisition and reinstatement of psychostimulant reward memory in mice. Behav. Brain Res..

[23] Hu SS, Liu YW, Yu L (2015). Medial prefrontal cannabinoid CB1 receptors modulate consolidation and extinction of cocaine-associated memory in mice. Psychopharmacology.

[24] Tung LW (2016). Orexins contribute to restraint stress-induced cocaine relapse by endocannabinoid-mediated disinhibition of dopaminergic neurons. Nat. Commun..

[25] Vaughn LK (2012). Cannabinoid receptor involvement in stress-induced cocaine reinstatement: Potential interaction with noradrenergic pathways. Neuroscience.

[26] Kupferschmidt DA, Klas PG, Erb S (2012). Cannabinoid CB1 receptors mediate the effects of corticotropin-releasing factor on the reinstatement of cocaine seeking and expression of cocaine-induced behavioural sensitization. Br. J. Pharmacol..

[27] McReynolds JR (2016). CB1 receptor antagonism blocks stress-potentiated reinstatement of cocaine seeking in rats. Psychopharmacology.

[28] Wiskerke J, Pattij T, Schoffelmeer AN, De Vries TJ (2008). The role of CB1 receptors in psychostimulant addiction. Addict. Biol..

[29] Floresco SB (2015). The nucleus accumbens: An interface between cognition, emotion, and action. Annu. Rev. Psychol..

[30] Chevaleyre V, Takahashi KA, Castillo PE (2006). Endocannabinoid-mediated synaptic plasticity in the CNS. Annu. Rev. Neurosci..

[31] Robbe D, Alonso G, Duchamp F, Bockaert J, Manzoni OJ (2001). Localization and mechanisms of action of cannabinoid receptors at the glutamatergic synapses of the mouse nucleus accumbens. J. Neurosci..

[32] Mahler SV (2014). Modafinil attenuates reinstatement of cocaine seeking: Role for cystine-glutamate exchange and metabotropic glutamate receptors. Addict. Biol..

[33] Moran MM, McFarland K, Melendez RI, Kalivas PW, Seamans JK (2005). Cystine/glutamate exchange regulates metabotropic glutamate receptor presynaptic inhibition of excitatory transmission and vulnerability to cocaine seeking. J. Neurosci..

[34] Volkow ND, Hampson AJ, Baler RD (2017). Don't worry, be happy: Endocannabinoids and cannabis at the intersection of stress and reward. Annu. Rev. Pharmacol. Toxicol..

[35] Morena M, Patel S, Bains JS, Hill MN (2016). Neurobiological interactions between stress and the endocannabinoid system. Neuropsychopharmacology.

[36] Percie du Sert N (2020). Reporting animal research: Explanation and elaboration for the ARRIVE guidelines 20. PLoS Biol..

[37] Clarke JR (2008). Posttraining activation of CB1 cannabinoid receptors in the CA1 region of the dorsal hippocampus impairs object recognition long-term memory. Neurobiol. Learn. Mem..

[38] Garcia-Keller C (2013). Cross-sensitization between cocaine and acute restraint stress is associated with sensitized dopamine but not glutamate release in the nucleus accumbens. Eur. J. Neurosci..

[39] Gomez R (2002). A peripheral mechanism for CB1 cannabinoid receptor-dependent modulation of feeding. J. Neurosci..

[40] Fuchs RA, Evans KA, Parker MC, See RE (2004). Differential involvement of the core and shell subregions of the nucleus accumbens in conditioned cue-induced reinstatement of cocaine seeking in rats. Psychopharmacology.

[41] Fuchs RA, Ramirez DR, Bell GH (2008). Nucleus accumbens shell and core involvement in drug context-induced reinstatement of cocaine seeking in rats. Psychopharmacology.

[42] Xie X (2012). Subregion-specific role of glutamate receptors in the nucleus accumbens on drug context-induced reinstatement of cocaine-seeking behavior in rats. Addict. Biol..

[43] Schmidt HD, Anderson SM, Pierce RC (2006). Stimulation of D1-like or D2 dopamine receptors in the shell, but not the core, of the nucleus accumbens reinstates cocaine-seeking behaviour in the rat. Eur. J. Neurosci..

[44] Robbe D, Kopf M, Remaury A, Bockaert J, Manzoni OJ (2002). Endogenous cannabinoids mediate long-term synaptic depression in the nucleus accumbens. Proc. Natl. Acad. Sci. U.S.A..

[45] De Vries TJ, Homberg JR, Binnekade R, Raaso H, Schoffelmeer ANM (2003). Cannabinoid modulation of the reinforcing and motivational properties of heroin and heroin-associated cues in rats. Psychopharmacology.

[46] Gamaleddin I (2012). Cannabinoid receptor stimulation increases motivation for nicotine and nicotine seeking. Addict. Biol..

[47] Fattore L, Spano M, Melis V, Fadda P, Fratta W (2011). Differential effect of opioid and cannabinoid receptor blockade on heroin-seeking reinstatement and cannabinoid substitution in heroin-abstinent rats. Br. J. Pharmacol..

[48] Biala G, Budzynska B (2008). Calcium-dependent mechanisms of the reinstatement of nicotine-conditioned place preference by drug priming in rats. Pharmacol. Biochem. Behav..

[49] Anggadiredja K (2004). Endocannabinoid system modulates relapse to methamphetamine seeking: Possible mediation by the arachidonic acid cascade. Neuropsychopharmacology.

[50] Daza-Losada M, Minarro J, Aguilar MA, Valverde O, Rodriguez-Arias M (2011). Acute blockade of CB1 receptor leads to reinstatement of MDMA-induced conditioned place preference. Pharmacol. Biochem. Behav..

[51] Hillard CJ (2014). Stress regulates endocannabinoid-CB1 receptor signaling. Semin. Immunol..

[52] Patel S, Roelke CT, Rademacher DJ, Hillard CJ (2005). Inhibition of restraint stress-induced neural and behavioural activation by endogenous cannabinoid signalling. Eur. J. Neurosci..

[53] Hill MN (2009). Suppression of amygdalar endocannabinoid signaling by stress contributes to activation of the hypothalamic–pituitary–adrenal axis. Neuropsychopharmacology.

[54] Patel S, Roelke CT, Rademacher DJ, Cullinan WE, Hillard CJ (2004). Endocannabinoid signaling negatively modulates stress-induced activation of the hypothalamic–pituitary–adrenal axis. Endocrinology.

[55] Gerdeman GL, Schechter JB, French ED (2008). Context-specific reversal of cocaine sensitization by the CB1 cannabinoid receptor antagonist rimonabant. Neuropsychopharmacology.

[56] Ahmed SH, Koob GF (1997). Cocaine- but not food-seeking behavior is reinstated by stress after extinction. Psychopharmacology.

[57] Stefanik MT, Kupchik YM, Kalivas PW (2016). Optogenetic inhibition of cortical afferents in the nucleus accumbens simultaneously prevents cue-induced transient synaptic potentiation and cocaine-seeking behavior. Brain Struct. Funct..

[58] Kerstetter KA (2016). Corticostriatal afferents modulate responsiveness to psychostimulant drugs and drug-associated stimuli. Neuropsychopharmacology.

[59] Bell K, Duffy P, Kalivas PW (2000). Context-specific enhancement of glutamate transmission by cocaine. Neuropsychopharmacology.

[60] LaLumiere RT, Kalivas PW (2008). Glutamate release in the nucleus accumbens core is necessary for heroin seeking. J. Neurosci..

[61] Suto N, Ecke LE, You ZB, Wise RA (2010). Extracellular fluctuations of dopamine and glutamate in the nucleus accumbens core and shell associated with lever-pressing during cocaine self-administration, extinction, and yoked cocaine administration. Psychopharmacology.

[62] Hotsenpiller G, Giorgetti M, Wolf ME (2001). Alterations in behaviour and glutamate transmission following presentation of stimuli previously associated with cocaine exposure. Eur. J. Neurosci..

[63] Miguens M (2008). Glutamate and aspartate levels in the nucleus accumbens during cocaine self-administration and extinction: A time course microdialysis study. Psychopharmacology.

[64] Suto N, Elmer GI, Wang B, You ZB, Wise RA (2013). Bidirectional modulation of cocaine expectancy by phasic glutamate fluctuations in the nucleus accumbens. J. Neurosci..

[65] Hoffman AF, Lupica CR (2001). Direct actions of cannabinoids on synaptic transmission in the nucleus accumbens: A comparison with opioids. J. Neurophysiol..

[66] Robbe D, Alonso G, Manzoni OJ (2003). Exogenous and endogenous cannabinoids control synaptic transmission in mice nucleus accumbens. Ann. N. Y. Acad. Sci..

[67] Kalivas PW, Lalumiere RT, Knackstedt L, Shen H (2009). Glutamate transmission in addiction. Neuropharmacology.

[68] Baker DA (2003). Neuroadaptations in cystine-glutamate exchange underlie cocaine relapse. Nat. Neurosci..

[69] Madayag A (2007). Repeated *N*-acetylcysteine administration alters plasticity-dependent effects of cocaine. J. Neurosci..

[70] Kalivas PW (2009). The glutamate homeostasis hypothesis of addiction. Nat. Rev. Neurosci..

[71] Sano K (2008). Delta 9-tetrahydrocannabinol-induced catalepsy-like immobilization is mediated by decreased 5-HT neurotransmission in the nucleus accumbens due to the action of glutamate-containing neurons. Neuroscience.

[72] Polissidis A (2013). The cannabinoid CB1 receptor biphasically modulates motor activity and regulates dopamine and glutamate release region dependently. Int. J. Neuropsychopharmacol..

[73] Li X (2018). mGluR5 antagonism inhibits cocaine reinforcement and relapse by elevation of extracellular glutamate in the nucleus accumbens via a CB1 receptor mechanism. Sci. Rep..

[74] Vasquez C, Lewis DL (1999). The CB1 cannabinoid receptor can sequester G-proteins, making them unavailable to couple to other receptors. J. Neurosci..

[75] Zhang LY (2021). Restoring glutamate homeostasis in the nucleus accumbens via endocannabinoid-mimetic drug prevents relapse to cocaine seeking behavior in rats. Neuropsychopharmacology.

[76] Navarrete M, Diez A, Araque A (2014). Astrocytes in endocannabinoid signalling. Philos. Trans. R. Soc. Lond. Ser. B Biol. Sci..

[77] D'Ascenzo M (2007). mGluR5 stimulates gliotransmission in the nucleus accumbens. Proc. Natl. Acad. Sci. U.S.A..

[78] Orellana JA (2015). Restraint stress increases hemichannel activity in hippocampal glial cells and neurons. Front. Cell. Neurosci..

[79] Kruyer A, Kalivas PW (2020). Astrocytes as cellular mediators of cue reactivity in addiction. Curr. Opin. Pharmacol..

[80] Schoffelmeer AN, Hogenboom F, Wardeh G, De Vries TJ (2006). Interactions between CB1 cannabinoid and mu opioid receptors mediating inhibition of neurotransmitter release in rat nucleus accumbens core. Neuropharmacology.

[81] Pertwee RG (2010). Receptors and channels targeted by synthetic cannabinoid receptor agonists and antagonists. Curr. Med. Chem..

[82] Baur R, Gertsch J, Sigel E (2012). The cannabinoid CB1 receptor antagonists rimonabant (SR141716) and AM251 directly potentiate GABA(A) receptors. Br. J. Pharmacol..

[83] Pickel VM, Chan J, Kash TL, Rodriguez JJ, MacKie K (2004). Compartment-specific localization of cannabinoid 1 (CB1) and mu-opioid receptors in rat nucleus accumbens. Neuroscience.

[84] Szabo B, Muller T, Koch H (1999). Effects of cannabinoids on dopamine release in the corpus striatum and the nucleus accumbens in vitro. J. Neurochem..

