# Supplementary material for: CB1R activation in nucleus accumbens core promotes stress-induced reinstatement of cocaine seeking by elevating extracellular glutamate in a drug-paired context
Source: Sci Rep. 2021 Jun 21;11:12964. doi: 10.1038/s41598-021-92389-4 (PMC8217548; doi:10.1038/s41598-021-92389-4)
Supplement: Supplementary file 1 — Supplementary Information. [file 41598_2021_92389_MOESM1_ESM.pdf]

CB1R activation in nucleus accumbens core promotes stress-induced reinstatement of cocaine seeking by elevating extracellular glutamate in a drug-paired context

Andrea S. Guzman, Maria P. Avalos, Laura N. De Giovanni, Pia V. Euliarte, Marianela A. Sanchez, Bethania Mongi-Bragato, Daiana Rigoni, Flavia A. Bollati, Miriam B. Virgolini, Liliana M. Cancela.*

Supplementary Materials and Methods

CPP Apparatus

The two-chambers apparatus consisted of two acrylic boxes (25cm x 33cm x 37cm) separated by a small corridor (22 cm x 11 cm x 19 cm). One of the main chambers was black with stainless steel grid floor; the other one was white with a plastic mesh floor. The central corridor was transparent with acrylic floor and separated from the two chambers by removable guillotine doors. The time spent in each chamber and locomotor activity were recorded using infrared photo beams (six beams for the main chambers and two beams for the central corridor) and a computer interface (LIADE, FCEyN, Córdoba, Argentina). The apparatus was placed in a semi-dark experimental room with only a single lamp illuminating all the apparatus, so both contexts were equally preferred by animals during the basal test (unbiased apparatus).

CPP General Procedure [1,2]

The CPP procedure consisted of the following phases: basal test (BT), conditioning phase, conditioning test (CT), extinction phase, extinction test (ET) and stress-induced reinstatement test (RT). The behavioral experiments were conducted during the light phase of the cycle between 10.00 AM and 4.00 PM. All animals were habituated to the experimental room for 30 min, in their home cages, before the beginning of each session in all phases. The BT (or preconditioning test) consisted of a single test in which each animal was placed into the central corridor with the guillotine doors removed, giving free access to the entire apparatus for 15 min. The amount of time spent in each chamber was recorded in order to exclude animals that showed unconditioned preference for either context (more than 66% of total time spent in main

chambers, basal exclusion criterion). It is important to note that all the following tests were performed in the same way, but with different criteria. During the conditioning phase, animals were confined for 30 min to one chamber (the less preferred in BT, either black or white indistinctly, biased assignment) immediately after receiving an injection of cocaine (10 mg/kg i.p.), or to the opposite chamber after receiving saline injection. After eight days alternating between drug/saline sessions (4/4 days), the CT was performed, allowing the animals to move freely through the entire apparatus for 15 min, in order to confirm the expression of the conditioned preference for the cocaine-paired context (more than 66% of total time spent in main chambers, conditioning inclusion criterion). The extinction phase consisted of eight successive, alternate saline associations (confinement for 30 min, identical to conditioning) with both contexts, in order to achieve a gradual disappearance of the cocaine-CPP, followed by the respective test (ET) at the end of this phase. If animals did not meet the extinction criterion (less than 66% of total time spent in the cocaine-paired context during the ET), they received two additional sessions as a maximum and an additional extinction test. Once animals reached the extinction criterion, they were submitted to microinjections and restraint stress on the RT day, in accordance to the experiment, before testing reinstatement of CPP. In general, on the RT day animals were first habituated to the experimental room for 30 min, as was done for every previous CPP sessions. Then, microinjections were performed as described below, and 5 min later animals were submitted to a restraint stress session (or stayed in their home cages for the same time period), and then immediately to a test. During the reinstatement test, animals had free access to the entire apparatus for 15 min and the amount of time spent in each chamber was recorded. The experimental groups are detailed in each figure and also in **Tables S1-S3**, showing the doses of CB1R ligands used in each experiment and the duration of restraint stress.

The schematic timelines for each experiment are presented in all the figures, including details of the minor modifications applied to this general procedure. The

behavioral experiments were analyzed taking into account the preference score, which is the time spent in cocaine-associated context minus the time spent in the saline-associated context (Fig.1 and 2), or the time spent in each compartment of the CPP apparatus (Fig. 5). These data are represented with bars in dot points for each test of the CPP procedure.

The percentage of animals excluded for BT, CT and ET was 6%, 30% and 5% respectively. Each percentage was calculated in relation to the number of animals that participated in each phase, i.e. in relation to the number of animals that met the inclusion criterion of the preceding test. Considering the initial amount of animals, 38% of animals were discarded.

Restraint Stress

On the RT day, rats were immobilized in a Plexiglas restraining device, which was placed into the central corridor of the CPP apparatus, 5 min after finishing the microinjection procedures. The flat-bottomed restrainer (half cylinders; 20 x 6 x 5 cm) contains several slits for air circulation and a removable plastic gate to hold the animal in the device with the tail emerging from the rear. For microdialysis experiments, with the aim of avoiding damage to the external parts of the probes, the upper half of the restraining device was modified in order to hold the rats down from the dorsal side instead of letting the animals enter the tube. The duration of the stress session was chosen according to previous results from our laboratory[1] and depending on the experiment: 30 min (S30), a reinstating stress session for the AM251 experiment, or 15 min (S15), a non-reinstating stress session for the ACEA experiment. The non-stressed (NS) group of rats was kept in their home cages during the same time period. Immediately after finishing the restraint, all animals were tested for reinstatement or were re-exposed to either compartment of the CPP apparatus for the *in vivo* microdialysis experiments. The experimental groups are detailed in each figure and also in **Tables S1-S3**, showing the duration of restraint stress used in each experiment.

Stereotaxic surgery procedures

Animals were anaesthetized with ketamine hydrochloride plus xylazine (55 mg/kg and 11 mg/kg, respectively, Kensol, Laboratories Holliday, Buenos Aires, Argentina). Animals were positioned in a stereotaxic frame (Stoelting Co, USA) with the incisor bar set at −3.3 mm relative to the inter-aural line. For the microinjection experiments, two stain-less steel guide cannula (22-gauge) were implanted bilaterally, 2 mm dorsal to the NAc. We targeted the NAcore or NAshell using the following stereotaxic coordinates (in mm from bregma, based on [3]): NAc Core: AP= +1.3; L= ±1.5; V= -4.3, NAc Shell: AP= +1.3; L= ±0.8; V= -4.4. For implantation of the microdialysis membrane in the NAcore, unilaterally, the coordinates were: AP= +1.3; L= ±1.5; V= -7.8. The guide cannula and the microdialysis probe were anchored to the skull with dental cement (Meron, Voco Laboratories, Cuxhaven, Germany) and skull screws. The cannula and dialysis membrane placements for each animal were confirmed after finishing the experiments.

For behavioral experiments, guide cannula implantations were performed after the fourth day of the extinction phase. Animals were allowed 2-3 days to recover from the surgery and then the extinction phase continued for the 4 remaining days.

For microdialysis experiments, animals were submitted to probe implantation the day before collecting samples. In this way, the following day after finishing the corresponding CPP test (depending on the experiment), stereotaxic surgery was conducted and animals were placed individually in their home cages allowing them to recover for at least 18 hours before proceeding with the microdialysis procedure.

Microinfusion Procedure

All drug infusions were performed inside the experimental room. For the microinfusions, rats were gently hand-held while the microinjector (30-gauge dental needle) was placed into the guide cannula, projecting 2 mm below its ventral end. Drug injections were conducted with a volume of 0.5 μl/side for one minute using an infusion pump (Harvard apparatus, USA) mounted with 5μl Hamilton syringes attached to

polyethylene (PE-20) tubing. The injector was left in place for an additional minute to facilitate diffusion of the drug before removal. All animals were previously habituated to the microinfusion procedure during the last two days of the extinction phase.

Microinjections were performed under reinstatement conditions, i.e. after extinction of cocaine-CPP. For behavioral experiments (Fig. 1 and 2), animals were first habituated to the experimental room for 30 min as was done for every CPP sessions, and then, microinjections were performed as described in the previous paragraph. Five minutes after finishing the microinjection, animals were submitted to a restraint stress session (or stayed in their home cages for the same time period), and immediately then to a test (RT). For the microdialysis experiments (fig. 3 and 4), microinjections were performed after collecting samples for baseline determination (see 'Microdialysis procedure' below). For this, animals were disconnected from microdialysis *tubing* to conduct a microinjection as described in the previous paragraph. Five minutes after finishing the procedure, animals were submitted to stress. The experimental groups are detailed in each figure and also in **Tables S1** and **S2**, showing the doses of CB1R ligands used in each experiment.

The selection of doses used for intra-NA administration was based on previous studies (0.001 or 0.01 fmol/0.5µl/side for ACEA[4] and 2.5 or 5 nmol/0.5µl/side for AM251[5]). AM251 was dissolved first in pure DMSO (50 and 100mM), followed by sterile saline, to a final concentration of 5 and 10mM (and injected at 2.5 or 5 nmol/0.5µl/side). In this way, the final dilution of DMSO was 10%, which was also used as a vehicle. ACEA was directly dissolved in sterile saline containing 0.1% DMSO. An exhaustive control of behavior and histology examination was performed in order to discard any possible effect of drugs and vehicle solutions.

Microdialysis probes

Two different types of microdialysis probes were constructed in our laboratory, according to the principal characteristics described in our previous work [6], in which it

has been developed a straightforward procedure to construct the dialysis probes following the Di Chiara design [7], with some minor modifications. In this study, the self-built probes were made using outer 22-gauge stainless steel cannulas and inner fused silica capillary tubings (inner diameter 50µm, outer diameter 150µm; Polymicro Technologies, Phoenix, AZ, USA). Each probe contained inlet and outlet ports and a semipermeable membrane attached and closed at the end of the probe circuit. The whole assembly was fixed with epoxy cement. The dialysis membrane (AN69HF, Hospal-Gambro, Meyzieu, France) had 2.0 mm of active dialyzing area and consisted of polyacrilonitril/sodium methalyl sulphonate copolymer with a molecular weight cutoff of 10kDa (inner diameter 240 µm, outer diameter 310 µm, wall thickness 50µm, with an average pore size of 29 A.). The dual microinjection-microdialysis probe had a guide cannula (22-gauge stainless steel needle) assembled to the inlet metal, through which the microinfusion of CB1R ligands was given, nearby the dialysis membrane.

Physical properties of CB1R ligands

The molecular weight of ACEA {Arachidonyl-2'-chloroethylamide hydrate, C22H36ClNO, Sigma-Aldrich} is 365.98 (anhydrous basis) and is soluble in ethanol. The work of Zheng et al. (2015)[8] also administrated ACEA locally (into dorsal hippocampus) via reverse dialysis.

The molecular weight of AM251 {1-(2,4-Dichlorophenyl)-5-(4-iodophenyl)-4-methyl-N-1-piperidinyl-1H-pyrazole-3-carboxamide, C22H21N4OCl2I, Sigma-Aldrich} is 555.24 and is not soluble in water.

In vitro recovery assay for microdialysis probes

An *in vitro* recovery assay was performed for all probes used for *in vivo* experiments with the aim of testing the recovery efficiency of our self-built microdialysis probes. The assay and the probe stereotaxic implantation were carried out on the same day. The procedure consisted in dipping the dialysis membrane into Ringer's solution (NaCl, 145 nM; KCl, 4.0 nM; $CaCl_2$, 2.2 nM; in purified water) which also

contained glutamate at a concentration of 40µM (Sigma Aldrich, St Louis, MO, USA). By connecting the probe with FEP Teflon microdialysis tubes (inner diameter 120µm, outer diameter 65µm OD; BASi, USA) and by using a syringe pump (BASi, USA) mounted with a 1ml Hamilton syringe, the dialysis membrane was perfused with Ringer's solution at a constant flow rate of 1.5 µl/min at room temperature (±22°C). After an equilibration time of 60 min, two successive samples were collected every 15 min. Immediately after, glutamate was quantified and the relative *in vitro* recovery was calculated by comparing the concentration of the sample with the known concentration measured for a sample taken from the solution into which the probe was dipped. On average, the i*n vitro* recovery percentage value for glutamate was approximately 10%, with values ranging from 5% to 15%. Since the *in vivo* microdialysis data was reported according to the baseline levels determined for each animal and since we determined that probes were not affected uniformly after the *in vivo* procedure, we did not apply a correction factor for glutamate values in experiments.

Microdialysis procedure

The day following probe implantation surgery, the animals state of health and probe conditions were checked before moving them to the experimental room (the same location where the CPP training was carried out). Once in the experimental room, awake animals were kept individually in their home cages for baseline determination. Microdialysis probes were connected with FEP Teflon microdialysis tubings and perfused continuously with Ringer's solution (the same used for the *in vitro* recovery determination), at a constant flow rate of 1.5 µl/min by using a syringe pump. Glutamate dialysate samples were automatically collected every 15 min in vials kept at 4 ºC. The length of inlet and outlet FEP tubings were equal for all animals and experiments. All samples were stored at −80°C until measurement.

After 120 min of collecting basal samples inside the home cage, rats were transferred to either compartment of the CPP apparatus, with the cocaine-paired or

unpaired context depending on the experiment. The objective was to determine the context-dependent changes in extracellular glutamate levels within NAcore during the re-exposure to the CPP apparatus, which were compared with the baseline levels. To carry out this, the dialysate samples were collected inside the compartment for one hour.

For pharmacological experiments under reinstatement conditions, animals were submitted to microinjections (through the guide cannula of the dual probe, see ‘Microinjection procedure’ above) and to restraint stress treatments before re-exposing the animals to the extinguished cocaine-paired context. In this case, it is important to mention that microdialysis tubings were disconnected from and reconnected to the probes before and after restraint stress, respectively. In the same way, the “no stress” group was also disconnected from, and reconnected to the dialysis perfusion system for the same time period in their home cages. Previous pilot experiments from our laboratory have demonstrated that for the vast majority of the cases this procedure did not affect the dialysis efficiency.

For different microdialysis experiments, each protocol for sample collection was drawn in figures. Changes in accumbal extracellular glutamate levels were reported as percent from baseline (% baseline). *Post-hoc* analysis were performed comparing the last sample of baseline with the first sample collected inside either compartment of the CPP apparatus.

The baseline was defined as the average concentration of the last four samples before exposure to different microinjection and stress treatments. Baseline determination was established when these consecutive samples differed by no more than 15% from each other. Data were discarded from the statistical analysis if subjects did not reach this criterion for baseline.

Sample collection was carried out not during restraint stress, but during re-exposure to the CPP apparatus, in accordance to the principal objective of this study

which was to evaluate changes in glutamate in the NAcore in the presence of drug-associated environmental cues.

Experimental groups for microdialysis experiments are detailed in each figure and in **Table S2** and **S3**.

Reverse microdialysis for ACEA administration

In animals that previously extinguished cocaine-CPP, the reverse microdialysis technique was performed in order to study the CB1R activation in conditions that allowed to explore the modulating effect on the basal extrasinaptic glutamate levels within NAcore. For that, the sample collection procedure was carried on similarly as described above. All the procedure was performed inside the experimental room with the lights on while rats stayed in their home cages, without any contact with the CPP apparatus and any other stimulus. Firstly, baseline was determined by collecting dialysates every 15 min for 2 hours by perfusing the probe implanted in NAcore with Ringer's solution (1.5 µl/min). Then, without disconnecting the circuit, the CB1R agonist ACEA, dissolved in Ringer's solution in a concentration of 10mM, was perfused through the dialysis probe during one hour. Consecutively, two additional concentrations of ACEA (100 and 1000 mM, solutions freshly prepared on day of the experiment) were administered in the same way. Thus, four 15-min microdialysis samples were obtained at each concentration of ACEA. With the reverse microdialysis technique, ACEA was continuously perfused into NAcore and the successive changes in extracellular glutamate levels induced by the CB1R activation could be calculated as a percentage of the baseline mean.

Glutamate quantification

Dialysates was assayed for glutamate content by reverse-phase HPLC coupled with electrochemical detection (ESA Coulochem III, Chelmsford, MA, USA). The mobile phase was composed of 100 mM Na_2HPO_4, 1.75% acetonitrile and 20% methanol; pH was adjusted to 6.67 with phosphoric acid (all HPLC-grade solvents were obtained

from Sintorgan S.A., Argentina). The purified water (polished water) used here for the preparation of the mobile phase and all analytical solutions was subjected to an extra purification treatment (polishing) in order to reduce the electrode background currents and provides better sensitivity in electrochemical detection.

The mobile phase was delivered by a pump (ESA Model 582 Solvent Delivery Module, Chelmsford, MA, USA) at a flow of 0.6 mL/min through a C18 reversed phase analytical column (Gemini N 3 μm; 150 × 4.6 mm; Phenomenex, USA). The concentration of glutamate was determined by precolumn derivatization with OPA/BME (O-phthalaldehyde and O-β-mercaptoethanol in sodium tetraborate buffer, pH 9.3), similarly to a previous work from our laboratory [6]. The working solution was prepared each day by diluting 250 μl of the OPA/BME solution in 750 μl of sodium tetraborate. For the analysis, 7.5 μl of the working solution was mixed with 10 μl of the microdialysis dialysate. After 2 min of reaction, 10 μl of samples was injected manually into the 20 μl injection loop of the HPLC system. Once the separation of sample compounds was achieved by reversed-phase column, glutamate was detected coulometrically (ESA Coulochem III, Bedford, MA, USA) using three electrodes: a guardcell (+650 mV); an oxidation analytical electrode (+150 mV); and a reduction analytical electrode (+550 mV; analytical cells ESA Model 5014B). The retention time and height of peaks of glutamate were compared with an external standard curved, calculated by measuring different concentrations of glutamate (1.25, 2.5, 5, 10, 20 and 40 μM) prepared in polished water and HPLC-grade methanol (50:50, v/v) [9]. Glutamate was quantified by using the PC integration software EZChrom Elite (an ESA Chromatography Data System).

Verification of Cannula Placement

To do the histological verification, after completion of experiments, animals were decapitated and brains were removed. Each brain was fixed in 4% paraformaldehyde solution prepared in 0.1M phosphate buffer. Later, 60 μm coronal sections at the NAc

level were generated using a Cryostat (CM1510-S, Leica). The sections were mounted on gelatin-coated slides and stained with cresyl violet. Representative photomicrographs are shown in **Fig. S1**. Animals with improper cannula placements or with excessive mechanical damage in the targeted site due to hemorrhage caused during surgery and injections, were excluded from the subsequent data analysis. The location of the probes and cannulas was reconstructed, and positioned referring to the Paxinos & Watson Atlas[3]. The approximate points of bilateral infusions into the NAc Core and Shell are shown in the each figure (1, 2, 3 and 4). The approximate membrane tracks of unilateral *probes* are shown in each figure (3, 4, 5, 6 and 7).

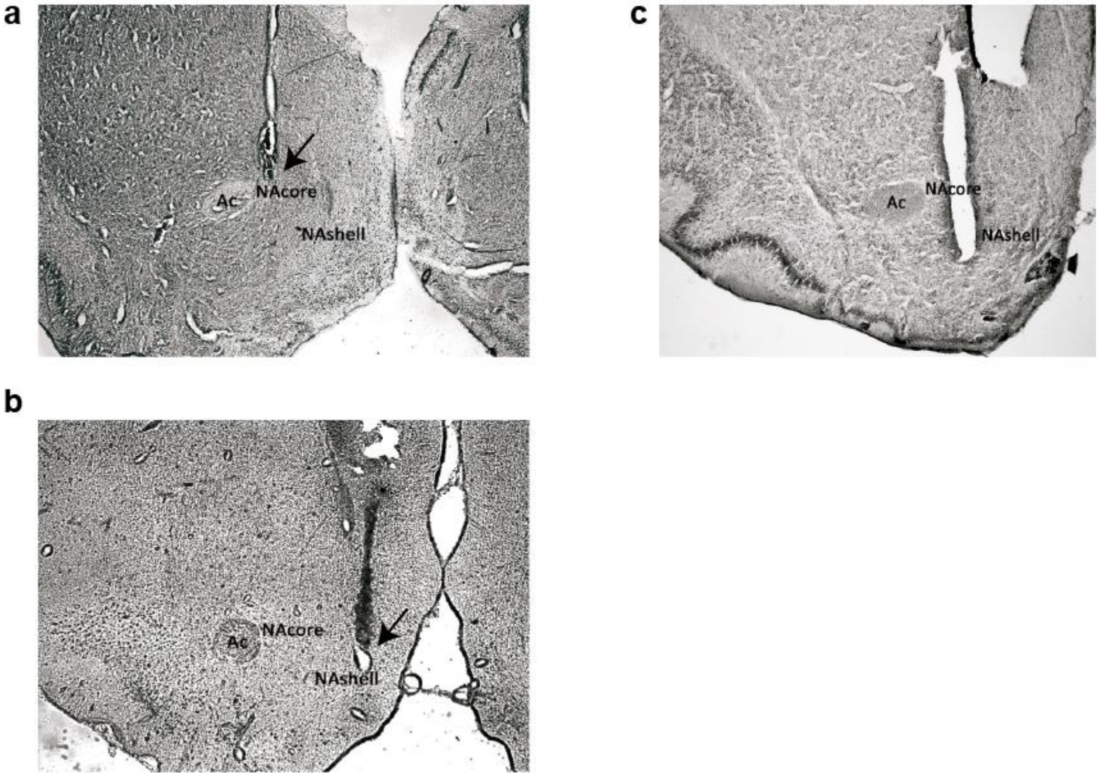

Figure S1. (a,b) Representative photomicrographs exemplifying the guide cannula tracks and the injection sites (arrows) of one rat that received microinjection into NAcore **(a)** and another that received the microinjection into NAshell **(b)**. **(c)** Representative photomicrograph exemplifying the microdialysis membrane track implanted in the NAcore.

Table S1. Experiments 1 and 2: Effects of CB1R antagonism and agonism in the NA on the restraint stress-induced reinstatement of cocaine-CPP.

Experiment	Results in figure…	Experimental Group	Treatments received in the RT day to evaluate stress-induced reinstatement			
			Local Microinjection (Pharmacological Treatment)			Restraint Stress
			Brain region	CB1R ligand	Dose	Duration (min)
1A	1b	VEH / NS	NAcore	Vehicle	0	0
		VEH / S30	NAcore	Vehicle	0	30
		AM251-2.5 / NS	NAcore	AM251	2.5 nmol/0.5ul/side	0
		AM251-2.5 / S30	NAcore	AM251	2.5 nmol/0.5ul/side	30
		AM251-5 / NS	NAcore	AM251	5 nmol/0.5ul/side	0
		AM251-5 /S 30	NAcore	AM251	5 nmol/0.5ul/side	30
1B	1d	VEH / NS	NAshell	Vehicle	0	0
		VEH / S30	NAshell	Vehicle	0	30
		AM251-5 / NS	NAshell	AM251	5 nmol/0.5ul/side	0
		AM251-5 /S 30	NAshell	AM251	5 nmol/0.5ul/side	30
2A	2b	VEH / NS	NAcore	Vehicle	0	0
		VEH / S15	NAcore	Vehicle	0	15
		ACEA 0.001/ NS	NAcore	ACEA	0.001 fmol/0.5ul/side	0
		ACEA-0.001 / S15	NAcore	ACEA	0.001 fmol/0.5ul/side	15
		ACEA-0.01/ NS	NAcore	ACEA	0.01 fmol/0.5ul/side	0
		ACEA-0.01 / S15	NAcore	ACEA	0.01 fmol/0.5ul/side	15
2B	2d	VEH / NS	NAshell	Vehicle	0	0
		VEH / S15	NAshell	Vehicle	0	15
		ACEA-0.01/ NS	NAshell	ACEA	0.01 fmol/0.5ul/side	0
		ACEA-0.01 / S15	NAshell	ACEA	0.01 fmol/0.5ul/side	15
2C	2f	VEH/ACEA-0.01 / S15	NAcore	Vehicle pretreatment + ACEA	0<br>0.01 fmol/0.5ul/side	15
		AM251 / ACEA-0.01 / S15	NAcore	AM251 pretreatment + ACEA	5 nmol/0.5ul/side<br>0.01 fmol/0.5ul/side	15

Table S2. Experiment 3 and 4: Effects of CB1R antagonism and agonism in the NAcore on the extracellular glutamate levels after exposure to restraint stress.

Experiment	Results in figure…	Experimental Group	Last CPP test applied	Treatments received in the microdialysis day, after baseline determination				Re-exposure to CPP context
				Local Microinjection (Pharmacological Treatment)			Restraint Stress	
				Brain region	CB1R ligand	Dose	Duration (min)	
3	3b	VEH / NS	ET	NAcore	Vehicle	0	0	Cocaine-paired
		VEH / S30	ET	NAcore	Vehicle	0	30	Cocaine-paired
		AM251-5 / NS	ET	NAcore	AM251	5 nmol/0.5ul/side	0	Cocaine-paired
		AM251-5 /S 30	ET	NAcore	AM251	5 nmol/0.5ul/side	30	Cocaine-paired
4	4b	VEH / NS	ET	NAcore	Vehicle	0	0	Cocaine-paired
		VEH / S15	ET	NAcore	Vehicle	0	15	Cocaine-paired
		ACEA-0.01/ NS	ET	NAcore	ACEA	0.01 fmol/0.5ul/side	0	Cocaine-paired
		ACEA-0.01 / S15	ET	NAcore	ACEA	0.01 fmol/0.5ul/side	15	Cocaine-paired

Table S3. Experiment 5: Context specific changes of extracellular glutamate in the NAcore throughout the CPP procedure.

Experiment	Results in figure…	Experimental Group	Last CPP test applied	Treatments received in the microdialysis day, after baseline determination		Re-exposure to CPP context
				Pharmacological Treatment	Restraint Stress	
5A	5c	Before conditioning	BT	No	No	Most preferred in BT
5B	5f	After conditioning, COC-unpaired	CT	No	No	Least preferred in CT
		After conditioning, COC-paired	CT	No	No	Most preferred in CT
5C	5i	After extinction	ET	No	No	Most preferred in CT
5D	6c	After extinction, COC unpaired	ET	No	Yes, 30 min	Least preferred in CT
		After extinction, COC paired	ET	No	Yes, 30 min	Most preferred in CT

Table S4. Experiment 6: Basal levels of accumbal extracellular glutamate regulated by CB1R agonism.

Experiment	Results in figure…	Experimental Group	Last CPP test applied	Treatments received in the microdialysis day, after baseline determination				Re-exposure to CPP context
				Pharmacological Treatment (by reverse microdialysis)			Restraint stress	
				Brain region	CB1R ligand	Increasing Doses		
6	7b	ACEA, Reverse microdialysis	ET	NAcore	ACEA	0, 10, 100, 1000 mM	No	No

References

1 De Giovanni, L. N., Guzman, A. S., Virgolini, M. B. & Cancela, L. M. NMDA antagonist MK 801 in nucleus accumbens core but not shell disrupts the restraint stress-induced reinstatement of extinguished cocaine-conditioned place preference in rats. *Behavioural brain research* **315**, 150-159, doi:10.1016/j.bbr.2016.08.011 (2016).

2 Mueller, D. & Stewart, J. Cocaine-induced conditioned place preference: reinstatement by priming injections of cocaine after extinction. *Behavioural brain research* **115**, 39-47, doi:10.1016/s0166-4328(00)00239-4 (2000).

3 Paxinos, G. & Watson, C. *The rat brain in stereotaxic coordinates*. 6th edn, (Academic Press/Elsevier, 2007).

4 Clarke, J. R. *et al.* Posttraining activation of CB1 cannabinoid receptors in the CA1 region of the dorsal hippocampus impairs object recognition long-term memory. *Neurobiology of learning and memory* **90**, 374-381, doi:10.1016/j.nlm.2008.04.009 (2008).

5 Xi, Z. X. *et al.* Cannabinoid CB1 receptor antagonist AM251 inhibits cocaine-primed relapse in rats: role of glutamate in the nucleus accumbens. *The Journal of neuroscience : the official journal of the Society for Neuroscience* **26**, 8531-8536, doi:10.1523/JNEUROSCI.0726-06.2006 (2006).

6 Garcia-Keller, C. *et al.* Cross-sensitization between cocaine and acute restraint stress is associated with sensitized dopamine but not glutamate release in the nucleus accumbens. *The European journal of neuroscience* **37**, 982-995, doi:10.1111/ejn.12121 (2013).

7 Di Chiara, G., Tanda, G., Frau, R. & Carboni, E. On the preferential release of dopamine in the nucleus accumbens by amphetamine: further evidence obtained by vertically implanted concentric dialysis probes. *Psychopharmacology* **112**, 398-402, doi:10.1007/bf02244939 (1993).

8 Zheng, L., Wu, X., Dong, X., Ding, X. & Song, C. Effects of Chronic Alcohol Exposure on the Modulation of Ischemia-Induced Glutamate Release via Cannabinoid Receptors in the Dorsal Hippocampus. *Alcoholism, clinical and experimental research* **39**, 1908-1916, doi:10.1111/acer.12845 (2015).

9 Donzanti, B. A. & Yamamoto, B. K. An improved and rapid HPLC-EC method for the isocratic separation of amino acid neurotransmitters from brain tissue and microdialysis perfusates. *Life Sci* **43**, 913-922, doi:10.1016/0024-3205(88)90267-6 (1988).
